# The forkhead transcription factor FOXK2 premarks lineage-specific genes in human embryonic stem cells for activation during differentiation

**DOI:** 10.1093/nar/gkaa1281

**Published:** 2021-01-12

**Authors:** Zongling Ji, Yaoyong Li, Sean X Liu, Andrew D Sharrocks

**Affiliations:** Faculty of Biology, Medicine and Health, University of Manchester, Michael Smith Building, Oxford Road, Manchester M13 9PT, UK; Faculty of Biology, Medicine and Health, University of Manchester, Michael Smith Building, Oxford Road, Manchester M13 9PT, UK; Faculty of Biology, Medicine and Health, University of Manchester, Michael Smith Building, Oxford Road, Manchester M13 9PT, UK; Faculty of Biology, Medicine and Health, University of Manchester, Michael Smith Building, Oxford Road, Manchester M13 9PT, UK

## Abstract

Enhancers play important roles in controlling gene expression in a choreographed spatial and temporal manner during development. However, it is unclear how these regulatory regions are established during differentiation. Here we investigated the genome-wide binding profile of the forkhead transcription factor FOXK2 in human embryonic stem cells (ESCs) and downstream cell types. This transcription factor is bound to thousands of regulatory regions in human ESCs, and binding at many sites is maintained as cells differentiate to mesendodermal and neural precursor cell (NPC) types, alongside the emergence of new binding regions. FOXK2 binding is generally associated with active histone marks in any given cell type. Furthermore newly acquired, or retained FOXK2 binding regions show elevated levels of activating histone marks following differentiation to NPCs. In keeping with this association with activating marks, we demonstrate a role for FOXK transcription factors in gene activation during NPC differentiation. FOXK2 occupancy in ESCs is therefore an early mark for delineating the regulatory regions, which become activated in later lineages.

## INTRODUCTION

The unique spatial and temporal patterns of gene expression during development are the result of the engagement of multiple enhancers regions which drive this carefully choreographed process (reviewed in 1). However, it is not clear how these enhancers are established, activated and decommissioned during cellular differentiation although pioneer transcription factors such as the FOXA subclass of forkhead (FOX) transcription factors are thought to be able to access the DNA while encapsulated in chromatin and initiate enhancer formation (reviewed in 2). Other studies have implicated additional FOX transcription factors in the enhancer commissioning process, including Foxh1 in Xenopus which marks and represses enhancers prior to mesendoderm specification (3) and FOXD3 which promotes chromatin opening while maintaining the underlying enhancers in a quiescent state in mouse embryonic stem cells (ESCs) and epiblast cells (EpiCs) prior to their activation in downstream lineages (4).

The FOX transcription factor family has over 40 members (reviewed in 5). While several FOX proteins have been implicated in enhancer activation, the role of the FOXK subclass members FOXK1 and FOXK2 remains unknown. In contrast to the restricted expression of most other FOX proteins, FOXK1/2 are ubiquitously expressed, suggesting a fundamentally important general role. Mechanistically, these proteins are generally thought to act in a repressive manner through binding to the co-repressor SIN3A and a range of other co-repressor proteins (6–13). This repressive activity of FOXK proteins has been shown to be crucial in suppressing autophagy programmes (14). However, more recent studies have begun to challenge this purely repressive function and suggest a potential role for both activation and repression of gene expression. For example, Foxk proteins promote the expression of genes encoding components from the glycolytic pathway and hence contribute to metabolic reprogramming (15,16). There is a growing appreciation of the biological importance of the FOXK proteins and their deregulation in diseases such as cancer (reviewed in 17). Developmentally, FoxK has been implicated in TGF-β signalling during midgut differentiation in *Drosophila* (18) and also has a role in stem cell biology as the mouse homologue of FOXK1, MNF, controls the proliferation of myogenic stem cells (8,19).

In this study, we investigated the role of FOXK2 during human embryonic stem cell (ESC) differentiation and in particular its impact on the regulatory chromatin environment. We find extensive FOXK2 binding to regulatory regions in ESCs, and although this binding is associated predominantly with active histone marks, it also pre-marks regulatory regions for future activation following differentiation. By focussing on neural precursor cells (NPCs), we demonstrate a role for FOXK transcription factors in promoting NPC differentiation. Importantly, enhancer regions prebound in ESCs show elevated activation in the transition to NPCs. In this context, we uncover a transcriptional activating role for FOXK proteins in addition to their well established repressive function.

## MATERIALS AND METHODS

### ESC culture, differentiation and stable cell line creation

H1 ESCs were grown in mTeSR1 (Stemcell Technologies) on Matrigel (BD Biosciences) coated culture dishes. Colonies were routinely passaged using ReLeSR (Stemcell Technologies) with the media changed daily. For mesendoderm (MESE) differentiation, H1 cells were cultured until 70–80% confluent, before dissociation using TryPLE (Gibco), and seeding at a density of 4 × 10^4^/cm^2^ in Matrigel coated culture plates, in mTeSR1 containing 5 ng/ml BMP4 (R&D systems, 314-BP-010) and 25 ng/ml Activin A (PeproTech 120-14E) for 40 h. For NPC differentiation, H1 cells were induced using STEMdiff™ Neural Induction Medium (Stemcell Technologies) using the monolayer culture method for 5 or 7 days with the media changed daily.

hESCs containing trimethoprim (TMP)-stabilised degron-tagged endogenous FOXK2 (H1-FOXK2-DHFR) was constructed by introducing the *Escherichia coli* dihydrofolate reductase (eDHFR) degron into the C-terminus of endogenous FOXK2 locus. Briefly, two targeting of gRNAs were designed (http://crispr.mit.edu) and ligated with pSpCas9(BB)-2A-GFP (PX458) (obtained from Addgene; plasmid # 48138) digested by BbsI (to create pAS4236 and pAS4237). A FOXK2-DHFR-Puro gBlock sequence was designed based on human FOXK2 genomic DNA and incorporating the HA-eDHFR-T2A-puro^R^ cassette (20), synthesised by Integrated DNA Technologies (IDT) and used as DNA donor for homology dependent repair. H1 cells were then co-transfected with pAS4236, pAS4237 and the FOXK2-DHFR-Puro gBlock DNA using FuGENE HD (Promega). Puromycin (1 μg/ml) was added 72 h after transfection, and the medium was changed daily. 10 μM trimethoprim (TMP, Sigma, 92131) was added to culture medium during transfection and selection. After 1–2 weeks, single colonies were selected, and genomic DNA was isolated using *DNeasy* Blood & Tissue Kits (Qiagen) for PCR genotyping. Controllable protein expression in FOXK2-DHFR cell lines was detected by Western Blot using whole cell lysates from cells treated with 10 μM TMP (+TMP) or DMSO (−TMP) for 24 h. The oligonucleotides and gBlock DNA sequence used to create and validate the FOXK2-DHFR cell line are listed in Supplementary Table S1).

### Flow cytometry analysis of NPC differentiation

For flow cytometric analysis of NPCs, cells were differentiated for 8 days and transfected by siRNA of FOXK1 and FOXK2 every 2–3 days. Cells were dissociated using TryPLE and washed with FACS buffer (1% BSA/PBS). Samples were stained for 30 min at 4°C with fluorochrome-conjugated CD15 (Stage-Specific Embryonic Antigen 1, SSEA1, BD Biosciences, 562369) or CD56 (Neural cell adhesion molecule, NCAM1, BD Biosciences, 561905) antibodies and washed 3 times using FACS buffer prior to analysis. Samples were stained using 0.25 μg/ml DAPI to exclude dead cells before collecting with BD LSR II flow cytometer (BD Biosciences) and analysed with Flowjo v10 software (BD Biosciences).

### RNA interference

siRNAs for FOXK1, FOXK2 and SIN3A were ON-TARGETplus SMART pools from Dharmacon (L-032790-01-0005, L-008354-00-0005 and L-012990-00-0020 respectively), and control non-targeting siRNAs (Dharmacon, D-001810-10-20) were used throughout. To carry out RNA interference (RNAi), cells were dissociated using TryPLE (Gibco) and transfected with 50 nM siRNA using Lipofectamine^®^ RNAiMAX (Invitrogen) according to the reverse transfection method in the manufacturer's instructions. For the first 24 h of transfection, cells were treated using the ROCK inhibitor LY27632 (Stemcell Technologies). 48 h after transfection or at the indicated times during differentiation processes, the cells were harvested for further analyses. To achieve knockdown over a 5-day period in NPCs, cells were treated for second time with siRNA 72 h after the first transfection.

### Western blot analysis and co-immunoprecipitation assays

Western blots derived from whole cell lysates or immunoprecipitated proteins were visualized after incubation with primary antibodies (listed in Supplementary Table S2) using IRDye infrared dye (Li-Cor Biosciences) conjugated secondary antibodies and the signal was collected with a Li-Cor Odyssey infrared imager.

Co-immunoprecipitation was performed as previously described (21), and anti-FOXK2 antibody (Bethyl Laboratories A301–729A) was used for immunoprecipitation. Whole cell lysates were from H1-FOXK2-DHFR ESCs cultured either in the presence of TMP (10 μM) in the media to maintain protein stability or in the absence of TMP to trigger FOXK2 degradation.

### RT-qPCR and RNA-seq analysis

Total RNA was isolated using a RNeasy kit (Qiagen) according to the manufacturer's instructions and transcripts detected in a one-step RT–qPCR reaction using Quantitect SYBR green reagent (Qiagen). The primer-pairs used for RT-qPCR experiments are listed in Supplementary Table S3. RNA samples were run in duplicate from three independent experiments. The housekeeping gene *HMBS* was used as an internal control to normalise the data. Statistical analysis for real-time PCR results was performed using the Student's *t-*test. The error bars in all graphs represent standard deviation.

RNA-seq was performed in triplicate. Paired-end reads were mapped to human genome hg19 by the aligner STAR with the two-pass option (22). A pair of reads were counted for one transcript if at least half length of both reads were mapped to the transcript. Differentially regulated genes between two different conditions were determined using the R package edgeR (23) with the criteria *P*-value <0.01 and the averaged transcripts per 10 million reads (TC10M) >1.

### Chromatin immunoprecipitation (ChIP)-qPCR and ChIP-seq assays

ChIP-qPCR and ChIP-seq analysis was performed essentially as described previously (21) with anti-FOXK2 antibody (Bethyl Laboratories A301-729A) or with a SOX2 antibody (Abcam, ab97959) as indicated. For ChIP-qPCR, 1 × 10^6^ to 5 × 10^6^ of 5-day differentiated NPC cells were used. Bound regions were detected by quantitative PCR (qPCR) (using primers listed in Supplementary Table S3), from three independent experiments. Statistical analysis for real-time PCR results was performed using the Student's *t*-test. The error bars represent standard error of the mean. 5 × 10^7^ cells were used for ChIP-seq experiments. Immunoprecipitated DNA was purified with a PCR purification kit (Qiagen) and ∼5 ng of DNA were processed for sequencing

### ChIP-seq data processing

For FOXK2 ChIP-seq analysis, two biological replicates were generated for each condition (i.e. H1 ESCs, mesendoderm cells, or neural precursor cells) and sequenced using the Illumina HiSeq4000 platform. The paired-end reads were aligned to the human genome hg19 using Bowtie2 (24) with the setting ‘very sensitive’ and all other default settings. The uniquely aligned reads were selected for the further analysis. First duplicate reads were removed using Picard (http://broadinstitute.github.io/picard/). Then the peaks were called from each of the two replicates with the corresponding input samples as control, using MACS2 (25) with the options ‘-g hs -f BAMPE -p 1e-6’.

The correlation coefficients between the two replicates of the read counts on the whole genome (i.e. on all the non-overlapping genomic regions of 1kb length in the genome except those regions overlapping with the ENCODE blacklist regions (26)) was between 0.85 and 0.95 for the two replicates in our ChIP-seq data. As the correlation coefficient was quite high between two replicates of each sample, the reads in the two replicates were therefore pooled, and with the pooled reads from the two input replicates as control, peaks were called again from the pooled ChIP-seq reads using MACS2 with the same settings as the above. The 54,805 FOXK2 peaks (H1 ESCs), 14 576 peaks (mesendoderm) and 67 451 peaks (NPCs) obtained from the pooled reads and which also overlapped with the peaks called from each of the two replicates were selected for further analysis.

We noted that the peaks identified by MACS2 often contain more than one subpeak. We therefore used an alternative approach by first locating the signal enriched regions (SERs) from ChIP-seq data, using HMM learning and then identifying all the subpeaks within SERs, using the software PeakSplitter (27). The HMM learning started with the aligned reads from the pooled biological replicates after the filtering processes described above. First the human genome was divided into non-overlapping windows of 200 bp, and the numbers of reads in each window were counted by overlapping the reads within each window. Then based on the read counts in those genomic windows a 4-state HMM model was learned and then the HMM model was applied to those windows to assign one of the four states to each window, using the software HMMseg (downloaded from http://noble.gs.washington.edu/proj/hmmseg/). The windows with the learned HMM state 3 or 4 were regarded as the signal enriched windows. We then moved the 200 bp windows by 100 bp and applied the same HMM learning process to those moved windows. The regions regarded as the signal enriched in both HMM models were used in the further analysis. We then applied the HMM models learned from the corresponding ChIP-seq sample to the input sample to identify the enriched windows in the input sample. Then we removed the signal enriched windows from the ChIP-seq sample which were also signal enriched windows in the input sample. We also filtered out the signal enriched windows if they overlap with any of the ENCODE DAC blacklisted regions. The final set of SERs were obtained from merging the filtered signal enriched windows. We then applied PeakSplitter to identify the subpeaks in SERs. We subsequently filtered these subpeaks by removing those whose normalised height (i.e. the number of reads per 10 M uniquely mapped reads at the summit of the subpeak) is less than 2. 115 134 subpeaks were selected in H1 ESCs, 101 770 peaks in mesendoderm cells and 175 684 peaks in NPCs.

To identify overlapping peaks between two samples, one peak was regarded as overlapping with another peak if they overlap by at least 30% of each of their lengths. Venn diagrams showing the overlapping of peaks from different ChIP-seq datasets were created using the R package Vennerable (https://github.com/js229/Vennerable). To filter this list for higher confident dataset-specific FOXK2 peaks, we applied further criteria: firstly, to select the subpeaks present in any particular cell type, we used a threshold of 2 on the normalised height of subpeaks in that cell type. In order to identify the subpeaks which are in one sample A but not in another sample B, we first calculated the normalised height in the sample B of the subpeaks identified in the sample A, and then computed the difference of the normalised height between the sample A and B, and finally applied a further normalisation step by dividing the height difference by normalised height in B (or to avoid large fold changes on low magnitude peaks, a value of ‘1’ was given to peaks with a value < 1). We then used the criteria of the final normalised difference >2 and no subpeak in dataset B within 100 bp of those subpeaks in dataset A to select the subpeaks in dataset A as those unique to A relative to B. The subpeaks in A which are regarded as being shared by A and B are those which are not unique to A relative to B as defined above and have subpeak in dataset B within 100 bp of their summits and the absolute difference between the normalised heights of the two summits respectively in datasets A and B <1. Any subpeaks which are neither unique to A relative to B (or vice versa) nor shared by A and B (as defined above) were excluded from the Venn diagrams.

### ChIP-seq data analysis

The genomic distribution of the ChIP-seq peaks were calculated using all the transcripts in the Ensembl human gene annotation database v75 with the human genome hg19. The genome was divided into five types of regions; promoter regions [–1 kb to 0.5 kb relative to TSS], putative enhancer regions [–20 kb to –1 kb relative to TSS], TTSs [–0.5 kb to 1 kb relative to TTS], gene body [TSS + 0.5 kb to TTS – 0.5 kb], and all other regions were classified as ‘distal intergenic’ regions. A peak was counted as in one region if its summit is located in the region.

To assign peaks to chromatin states, we downloaded the 15-state ChromHMM data on H1 cells, which segmented the human genome into 15 types of regions such as ‘Active TSS’, ‘Enhancer’ and ‘Quiescent/Low’ by a HMM model learned from H1’s five chromatin marks (28). We then counted the numbers of FOXK2 peaks which overlap by at least 30% with each of those ChromHMM regions and plotted the bar graphs to show the distribution of the peaks across these different categories.

We also downloaded the three histone marks, H3K4me1, H3K27ac, and H3K27me3 of the five types of cells, H1 ESC, H1 derived mesendoderm, and HUES64 derived ectoderm, endoderm, and mesoderm from the Roadmap Epigenomics web site (http://egg2.wustl.edu/roadmap/data/byFileType/alignments/consolidated/). We then ran the chromHMM (29) to learn an HMM models of 6 states from the downloaded histone marks of the five cell types and then applied the HMM models to each cell type to assign HMM states to all the genomic regions for that cell type. We then overlapped the FOXK2 peaks with the genomic regions of different HMM states to calculate the distribution of the FOXK2 peaks in the six HMM states in one cell type.

In order to assess the correlation between FOXK2 binding and the presence of other histone marks, we first downloaded the ChIP-seq data for histone marks in H1 ESCs from the 111 consolidated epigenomes datasets of the Roadmap Epigenomics project (28). The ChIP-seq datasets of SIN3A, SAP30 and HDAC2 of H1 cells were downloaded from the ENCODE web site (30) (https://www.encodeproject.org/) with the following identifiers: ENCFF526QOE (Michael Snyder, Stanford), ENCFF258PMU (Bradley Bernstein, Broad), and ENCFF461HDT (Bradley Bernstein, Broad). In order to assess the correlation between two ChIP-seq samples, we first divided the whole genome into non-overlapping 1 kb sections, and removed those sections overlapping with the ENCODE DAC blacklisted regions or with the regions with mappability score ≤0.2 from the ENCODE CRG alignability 100mer data. Then for each sample we calculated the number of reads on each section. Finally, using the read counts found in each section, we computed the pairwise Pearson correlation coefficients between each two samples.

In order to compare the magnitudes of the histone marks between FOXK2 and SIN3A in H1 ESCs, we used the 54 805 FOXK2 peaks obtained by MACS2 from our ChIP-seq data and the 21,309 SIN3A peaks downloaded from the ENCODE web site. We first determined the peaks shared by both factors by taking FOXK2 peaks which overlap by at least 30% with the SIN3A peaks. The remainder were classified as either FOXK2 or SIN3A alone peaks. Then we calculated the averaged read counts of the normalised Roadmap histone mark data on these FOXK2 or SIN3A peaks (FOXK2 peaks were used for the overlapping peaks), which were used to draw the boxplots for comparing the different histone modification signal on the three sets of peaks. The lower and upper boundaries of the boxes denote the first quartile and the third quartile, and the whiskers extend to the most extreme data point which is no more than 1.5 times the interquartile range from the box.

Enrichment scores for overlaps of the FOXK2 peaks with H3K27ac peaks found in ESCs as they differentiate to NPCs over a 72-h period were calculated by first counting the number of the peaks in one set overlapping with the peaks in another set, then dividing the overlap number by the number of peaks in each of the two sets, and finally multiplying the number by 10 000 in order to avoid a too small value.

To identify potential transcription factor binding sites, motif enrichment analysis was performed on the 101 bp windows centred at the summit of the FOXK2 ChIP-seq peaks, using the software Homer (31) with the default settings.

In order to investigate whether FOXK2 or SIN3A binding was associated with the expression levels of the nearby genes, we focussed on high confidence assignments by associating ChIP-seq peaks to genes if their promoter regions (–1 kb to 0.5 kb relative to TSS; Ensembl human gene database v75) include at least 30% of the peak length. We obtained the expression level of the genes in H1 cells from the Roadmap Epigenomic data (28). We then created boxplots to show the expression distribution of the genes in the different categories.

To draw an average tag density plot of several samples over a selected set of genomic regions, we first obtained the number of reads of one sample at each nucleotide position in the regions by using the software seqMINER (32). We normalised the numbers of reads by the total mapped reads of the sample as per 10M reads. We then aligned the genomic regions (e.g. aligning the peak regions by peak summit) and averaged the numbers over the regions to obtain the averaged tag profile over a set of genomic regions. Finally the R plot functions were used to draw the density plot (https://www.R-project.org).

To associate FOXK2 binding events with potential biological functions, we used the online tool GREAT (33) to obtain the GO terms for each set of ChIP-seq peaks. Then for each set of GO terms we used another online tool REVIGO (34) to simplify (i.e. removing one term if it is very close to another term semantically), visualise and cluster these GO terms. Each cluster is identified by using the name of a representative term and visualised in a 2D scatter plot created in semantic space using semantic distances between the terms and multidimensional scaling.

### Statistical analysis

To compare the histone modification ChIP-seq signals on regions co-bound by FOXK2 and SIN3A, *P*-values were calculated on the resulting boxplots using a *t*-test. However, this indicated that, due to the large sample sizes (i.e. the number of peaks in each case) everything was significantly different between groups. This meant that any difference (even very small) between the means of the two compared groups scored as significant. However, we corrected for this by using a commonly used effect size index, the Cohen's *d* (35). We reported this using the commonly used criteria: *d* < 0.2 = no difference; 0.2 < *d* < 0.3 = small difference; 0.3 < *d* < 0.8 = medium difference; *d* > 0.8 = large difference.

### Datasets

Our ChIP-seq and RNA-seq data have been deposited with ArrayExpress. Accession numbers: FOXK2 ChIP-seq in ESCs, MESE cells and NPCs (E-MTAB-9630). FOXK1/K2 and SIN3A siRNA RNA-seq experiments in ESCs and FOXK1/2 siRNA experiments in differentiating NPCs (E-MTAB-9639). Previously published datasets are shown in Supplementary Table S4.

## RESULTS

### FOXK2 co-associates with active chromatin regions in human ESCs

**Figure 1.**
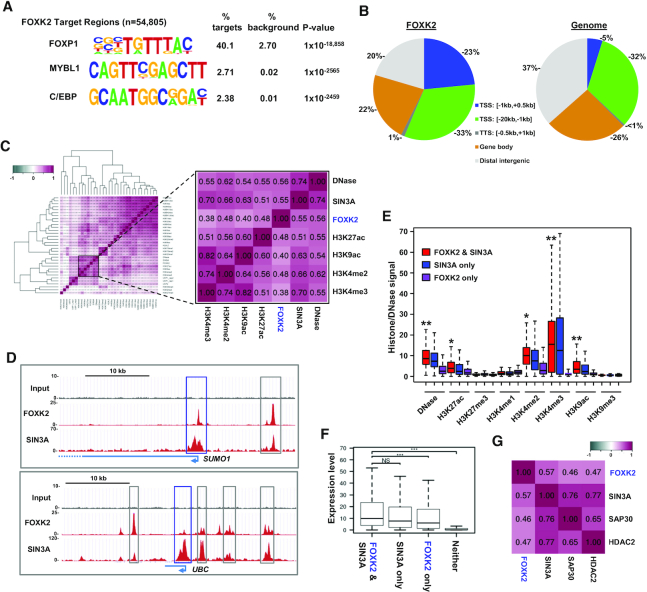
Characterisation of FOXK2 binding regions in human ESCs. (**A**) *De novo* motif discovery in FOXK2 binding regions. The closest matches of catalogued motifs to the discovered motifs are indicated. (**B**) Distributions of FOXK2 binding regions across different genomic locations (left) compared to the genome-wide frequency (right). (**C**) Hierarchical clustering of Pearson's correlation coefficients between ChIP-seq data for FOXK2, SIN3A and the indicated histone modifications (28). Datasets closely related to FOXK2 are highlighted. (**D**) UCSC genome browser view of FOXK2 and SIN3A ChIP-seq binding profiles around the *SUMO1* and *UBC* loci. Proximal promoter (blue) and intragenic (grey) regions co-bound by FOXK2 and SIN3A are boxed. (**E**) Boxplots of ChIP-seq signals for DNase sensitivity or the indicated chromatin marks at regions co-bound by FOXK2 and SIN3A or either factor alone. Asterisks represent *P*-values <0.001 and passing the effect size Cohen's *d* test as a large (**; score *d* >0.8) or medium (*; score 0.3 < *d* < 0.8) difference in signal between FOXK2 alone and FOXK2 and SIN3A co-binding. Horizontal line represents median expression and whiskers extend to the most extreme data point which is no more than 1.5 times the interquartile range from the box. (**F**) Boxplots of RNA expression levels of the genes associated with regions co-bound by FOXK2 and SIN3A or either factor alone. Horizontal line represents median expression and whiskers extend to the most extreme data point which is no more than 1.5 times the interquartile range from the box. ***P-value <0.001. (**G**) Pearson's correlation coefficients between ChIP-seq data for FOXK2 and the indicated SIN3A co-repressor complex components.

To begin to understand the role of FOXK2 in human ESCs we first determined its genomic binding locations in human H1 cells by ChIP-seq analysis. The intersection of two replicate experiments identified 54 805 FOXK2 binding regions. FOXK2, like other FOX transcription factors, binds to the core GTAAACA (inverse TGTTTAC) motif and both known motif analysis (Supplementary Figure S1A) and *de novo* motif analysis (Figure 1A) identified the expected FOXK2 transcription factor binding motif (denoted as FOXP1) in >40% of all binding regions, demonstrating the high quality of this dataset. This motif was found at high frequency (>30%) across the majority of peaks irrespective of peak ranking (Supplementary Figure S1B). However, although several other transcription factor motifs are significantly enriched, their very low frequency does not support the existence of a common co-regulatory module or the possibility of alternative binding motifs for FOXK2. FOXK2 exhibits a genome-wide distribution that is skewed towards proximal promoter regions at the expense of distal intergenic regions (Figure 1B). We next asked how many of these regions overlap with FOXK2 binding in U2OS osteosarcoma cells (21). A high degree of overlap is observed (35%) and this overlap is particularly high within promoter regions (51%) and lower in distal intergenic regions (Supplementary Figure S1B). A large number of FOXK2 binding events are therefore likely established in human ESCs and subsequently maintained, especially in promoter proximal regions.

Next, to determine whether any chromatin features are associated with FOXK2 binding regions, we examined correlations between FOXK2 binding events and other ChIP-seq datasets for a range of histone modifications in H1 cells (Figure 1C; Supplementary Figure S1D). The known interaction partner SIN3A (9,11) was included as a control and interacts with FOXK2 in hESCs alongside HDAC1 and BAP1 (Supplementary Figure S2A). As expected, FOXK2 binding shows high correlation with SIN3A (Figure 1C) with co-binding observed in both promoter and enhancer regions (Figure 1D; Supplementary Figures S2B, S3, S4 and S5A). However, FOXK2 binding is also highly correlated with open chromatin (DNase accessibility) and a range of histone marks that are associated with transcriptional activation, including H3K4me3 that is typically found at active promoters (36) and H3K27ac that is found at active promoters and enhancers (37) (Figure 1C; Supplementary Figures S2, S3 and S4). In contrast, there is very little enrichment of the repressive histone marks H3K9me3 and H3K27me3 at FOXK2 binding loci (Figure 1E; Supplementary Figures S2–S4). This finding was somewhat unexpected given that FOXK2 has been shown to be associated with multiple corepressor complexes (9,11) including SIN3A which is generally considered to be involved in transcriptional repression (38). We therefore examined whether SIN3A occupancy at the TSS correlated with gene expression levels and found that SIN3A occupancy increased as the level of gene expression increased (Supplementary Figure S5A), suggesting a role associated with gene activation. To extend these observations, we examined whether the co-occurrence with SIN3A on chromatin marks less or more active FOXK2 binding regions. However on the contrary to a predicted repressive effect, co-binding of SIN3A and FOXK2 is associated with higher levels of activating marks than either factor alone (Figure 1E; Supplementary Figure S4) and co-binding is also strongly enriched at regions defined as active promoters in H1 cells (28; Supplementary Figure S6). We further explored this relationship by investigating the activity of genes associated with FOXK2 and found that co-association with SIN3A is associated with higher gene expression in H1 cells than genes occupied by FOXK2 alone (Figure 1F). Thus FOXK2 and SIN3A are associated with active chromatin regions. SIN3A is known to associate with other proteins to form a co-repressor complex, including SAP30 and HDAC2. These proteins also show strong co-association with FOXK2 (Figure 1G) ruling out the possibility that FOXK2 associates with SIN3A in the absence of these co-repressor partner proteins. Previous work implicated TET1 as a co-activator for SIN3A in mouse ESCs (39). We therefore asked whether the co-bound FOXK2-SIN3A regions also associate with TET1 in human ESCs (40) and found a highly significant overlap (Supplementary Figure S5B). Furthermore, the promoter regions triply bound by FOXK2, SIN3A and TET1 are associated with more highly expressed genes than those bound by FOXK and SIN3A alone (Supplementary Figure S5C). This further emphasises the association of FOXK2 with gene activation and demonstrates that co-association with SIN3A and TET1 marks genes for transcriptional activation.

Collectively, these results demonstrate widespread binding of FOXK2 in the genome of H1 ESCs that is generally associated with active chromatin and co-associated active genes. Frequent co-binding with its partner protein SIN3A occurs at these active chromatin regions.

### Dynamic changes to FOXK2 chromatin binding accompany mesendodermal differentiation

**Figure 2.**
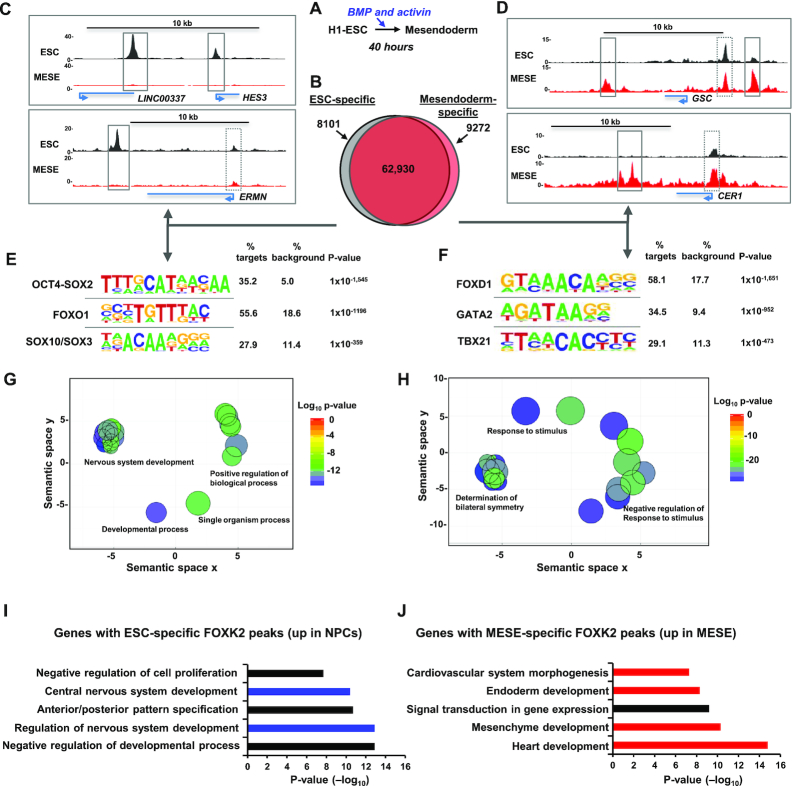
Identification of cell type-specific FOXK2 binding events. (**A**) Schematic view of the mesendoderm differentiation protocol. (**B**) Venn diagram showing the overlap of FOXK2 binding regions in ESCs and mesendodermal (MESE) cells. (**C** and **D**) UCSC genome browser view of FOXK2 ChIP-seq binding profiles in ESCs or MESE cells around the indicated loci. Cell type-specific (solid line) and common (dotted line) regions bound by FOXK2 are boxed. (**E** and **F**) De novo motif discovery in ESC-specific (E) and MESE-specific (F) FOXK2 binding regions. (**G** and **H**) Gene ontology enrichments for genes associated with ESC-specific (F) and MESE-specific (H) binding regions. GO terms are reduced and clustered in semantic space based on their semantic similarities and a single representative GO term is selected for each cluster, using the online tool REVIGO. (**I**) and (**J**) Gene Ontology categories enriched in genes associated with ESC-specific (I) or MESE-specific (J) FOXK2 peaks and showing upregulation (>2-fold change and >10 RPKM) in either NPCs (I) or MESO (J) cells. Data are ranked according to *P*-values.

The results from H1 ESCs suggest a role for FOXK2 in the context of an active chromatin environment. To establish whether this is the case in other cells and whether this changes as cells adopt different fates, we differentiated H1 ESCs to mesendodermal cells (MESE) by culturing in BMP4 and Activin A for 40 h (41,42) (Figure 2A). The cells exhibited the expected expression of mesendodermal markers (e.g. T brachyury and EOMES) and a loss of pluripotency markers (eg SOX2) (Supplementary Figure S7). ChIP-seq was performed in these cells, and replicate datasets combined before peak calling for comparison with FOXK2 binding regions in ESCs. We noticed that FOXK2 peaks were often clustered together with multiple sub-peaks (see Figure 1D), therefore to identify peaks that were unique to each cell type, we recalled peaks in ESCs and MESE cells by selecting for subpeaks by using PeakSplitter (27). There is a high congruence of FOXK2 binding in these cell types with 89% of FOXK2 peaks in ESCs maintained in MESE cells (ESC&MESE) (Figure 2B). However, we also detected 8101 FOXK2 peaks that are lost (ESC-specific) and 9272 peaks that are gained upon differentiation into MESE cells (MESE-specific) (Figure 2B–D). In both cases, cell type-specific FOXK2 binding is enriched at distal intergenic and intragenic regions while the frequency of promoter-proximal binding events decrease compared to the regions commonly bound in both cell types (Supplementary Figure S8A).

Motif analysis identified the expected FOX protein binding motifs in both the ESC-specific and MESE-specific binding regions (Figure 2E and F; denoted as FOXO1 and FOXD1 respectively). However, several other transcription factor motifs are strongly enriched in both datasets. In the ESC-specific regions, there is enrichment of composite OCT4-SOX2 elements, consistent with the loss of pluripotency following differentiation. However, there is also enrichment of SOX binding motifs, and these factors are known to play a role in both ESCs and in differentiation towards the neuronal lineage (43). Conversely, in MESE-specific peaks, there is enrichment of GATA factor and T-box protein binding motifs, which is consistent with the role of regulators like GATA4 and GATA6 (44) and T-brachyury (45) in promoting mesendodermal differentiation. In contrast, in the shared regions, only FOX binding motifs were enriched with both high frequency and probability (Supplementary Figure S8B).

We also associated the cell type-specific binding regions with nearby genes and asked whether these gene sets are associated with any particular biological processes. The genes associated with ESC-specific FOXK2 binding regions are associated with processes centred on neuronal differentiation and brain development (Figure 2G; Supplementary Figure S8C). A different set of biological processes were identified for the MESE-specific FOXK2 associated genes, with a large number of terms associated with developmental processes typical of the mesendodermal lineage, including symmetry determination, gastrulation, and heart development (Figure 2H; Supplementary Figure S8D). We also applied more stringent criteria and only considered MESE-specific FOXK2 binding regions that were associated with genes whose expression increased in MESE cells. These genes are associated with GO terms related to heart and endoderm development (Figure 2J). We also applied the same analysis to FOXK2 binding regions uniquely found in ESCs and investigated whether there was evidence for upregulation of associated genes in neuronal progenitor cells (NPCs). When looking at this subset of FOXK2 binding regions, GO terms were identified associated with various elements of neuronal development (Figure 2I).

These results therefore demonstrate a dynamic change in the FOXK2 binding landscape as ESCs differentiate down the mesendodermal lineage. FOXK2 binding events are both lost and gained. The sites which are lost are characterised by association with genes involved in neuronal developmental and contain binding motifs for transcription factors that are linked with this process. Conversely, those which are gained are associated with genes involved in different developmental processes and contain binding motifs for transcription factors that drive the development of mesendoderm and developmental processes downstream from this lineage.

### Dynamic changes to FOXK2 chromatin binding are associated with changes in chromatin and gene activation profiles

**Figure 3.**
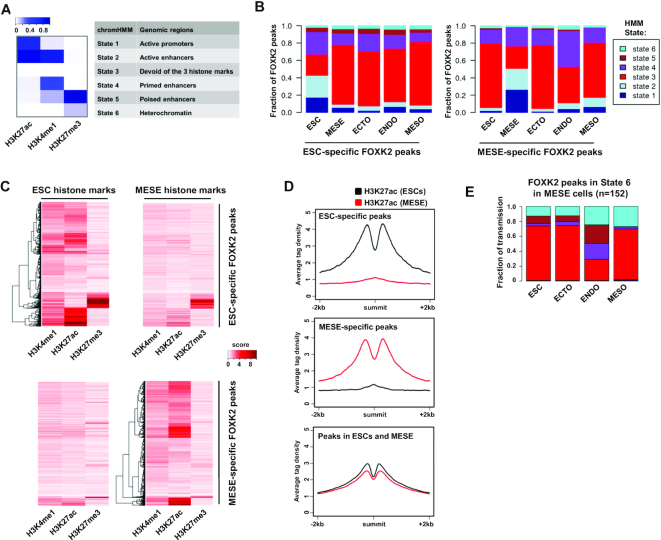
Fate of ESC-specific FOXK2 peaks during differentiation. (**A**) Heatmap showing six chromHMM chromatin states found using H3K27ac, H3K4me1 and H3K27ac data in H1 ESC, MESE, ECTO, ENDO and MESO cells. The colour scale bar represents the emission probabilities of the histone marks in the learned HMM model. (**B**) HMM states for regions containing the indicated categories of FOXK2 peaks in ESCs and the indicated differentiated cell types (MESE = mesendoderm; ECTO = ectoderm; ENDO = endoderm; MESO = mesoderm). (**C**) Heatmap showing the relative read density of the indicated histone modifications around the indicated subclasses of FOXK2 peaks. Data are show for histone marks in ESCs (left) or MESE cells (right). Data were normalised numbers of reads overlapping with peaks, with a score capped at a value of 10. (**D**) Average tag density plots of H3K27ac levels from either ESCs (black line) or MESE cells (red line) in a 4 kb window around the summit of the indicated categories of FOXK2 peaks. (**E**) HMM state transitions for MESE-specific FOXK2 peaks found in state 6 in MESE cells. The fates of these regions in ESCs and the indicated differentiated cell types are shown.

The identification of FOXK2 binding regions that are unique to a particular cell type was intriguing, suggesting a potential dynamic role in either modifying or responding to their local chromatin environment. We therefore studied the chromatin status of FOXK2 binding regions and how these change during differentiation from ESCs to various cell lineages. First we partitioned the genome into six different chromatin states using published data (28) and a hidden Markov model (HMM) (29) based on three histone marks in either ESCs or cell types derived from these (Figure 3A). We then determined the distribution of FOXK2 sites overlapping with each of these states (Figure 3B). The ESC-specific FOXK2 binding regions are fairly evenly distributed among states 1–4 in ESCs reflecting its association with active promoters (state 1), active and primed enhancers (states 2 and 4) but also regions devoid of histone marks (state 3). Generally, these FOXK2-bound regions reverted to state 3 (ie a loss of all histone marks) upon differentiation with ectodermal cells retaining the highest level of state 4 which is characterised by high levels of H3K4me1, an enhancer priming mark. Similarly, the MESE-specific FOXK2 binding regions are distributed among states 1–4 in MESE cells but are found in state 3 in other cell types with the exception of endodermal (ENDO) cells where a high proportion of state 4 (primed enhancers) is retained. In contrast, the distribution among chromatin states of FOXK2 binding regions shared between ESCs and MESE cells is relatively even across different cell lineages (Supplementary Figure S9A). Thus, in both cases, the cell type-specific FOXK2 binding regions generally show decommissioning from active histone marks with retention of the enhancer priming mark H3K4me1 in the immediate downstream cell lineage (ie endodermal cells in the case of MESE-specific FOXK2 peaks). To further investigate this, we compared the levels of each histone mark around the cell-type specific peaks. ESC-specific FOXK2 binding regions showed a general loss of H3K27ac and H3K4me1 upon conversion to MESE cells (Figure 3C and D; Supplementary Figure S9C and D). Conversely, MESE-specific FOXK2 peaks showed a general increase of H3K27ac and H3K4me1 upon differentiation to MESE cells from ESCs (Figure 3C and D; Supplementary Figure S9C and D). In contrast, FOXK2 binding regions shared between ESCs and MESE cells showed little change in the levels of these two histone marks in the two cell types. (Figure 3D; Supplementary Figure S9B and C). These data therefore strengthen the links between FOXK2 binding and active chromatin regions, and suggest a possible role in either promoting the formation of active regions and/or their subsequent maintenance and activity. The lack of association with the repressive H3K27me3 repressive mark further emphasises this point (Figure 3C; Supplementary Figure S9D).

We did however notice that there are a relatively small number of FOXK2 binding regions associated with chromatin state 6 (i.e. marked with repressive H3K27me3). One possible role of FOXK2 binding at these regions might be to prime for downstream activating events. We therefore examined the fate of this subclass of FOXK2 binding regions found in MESE cells in other lineages. Generally these regions are either retained in state 6 or converted to state 3 and become devoid of histone marks. However, in endodermal (ENDO) cells, there is a high degree of conversion to states 4 and 5 which are characterised by H3K4me1, and hence potential enhancer priming (Figure 3E). This result is consistent with a model in which FOXK2 binding occurs in MESE cells at inactive chromatin regions and these regions are primed for activation during subsequent differentiation events (in this case mesendoderm to endoderm).

### FOXK2 chromatin binding dynamics in neuronal precursor cells

Our discovery that FOXK2 binding is dynamic and changes during differentiation to MESE cells also uncovered a potential role for FOXK2 binding to regulatory regions in ESCs that are primed for later activation in NPCs. One prediction of this model is that FOXK2 binding events that are apparently specific to ESCs, i.e. not retained in the MESE lineage, would be retained upon differentiation to NPCs. We therefore performed ChIP-seq for FOXK2 following differentiation of ESCs to NPCs. This approach would also enable us to identify potentially novel NPC-specific peaks for FOXK2 binding which would further test the dynamic nature of the FOXK2 cistrome.

**Figure 4.**
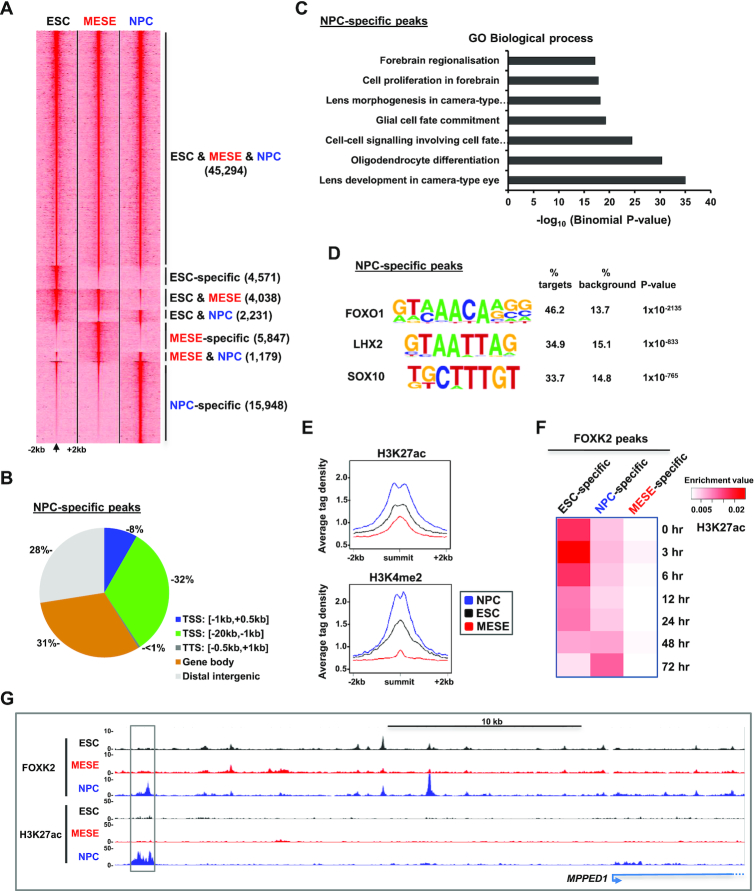
Genome-wide binding of FOXK2 in NPCs. (**A**) Heatmap of tag densities of FOXK2 binding at genomic loci in ESCs, NPCs and MESE cells. (**B**) Distributions of NPC-specific FOXK2 binding regions across different genomic locations. (**C**) Gene Ontology categories enriched in genes associated with NPC-specific FOXK2 peaks. (**D**) Motif discovery in NPC-specific FOXK2 binding regions (±200 bp from peak centre). (**E**) Average tag density plots of H3K27ac or H3K4me2 surrounding the summits (±2 kb) of the NPC-specific FOXK2 binding peaks. Data are shown for histone marks from NPCs (blue), ESCs (black) or MESE cells (red). (**F**) Overlaps between FOXK2 peaks in each indicated category and peaks of H3K27ac at the indicated time points of differentiation towards NPCs (59). Data are normalized for the numbers of peaks in each dataset. (**G**) UCSC genome browser views of FOXK2 and H3K27ac ChIP-seq binding profiles in the indicated cell types at the *MPPED1* locus. An NPC-specific FOXK2 binding peak associated with NPC-specific H3K27ac is boxed.

We differentiated H1 ESCs down the neuronal lineage to NPCs and these cells exhibited the expected expression of NPC markers (eg LHX2 and PAX6) and a loss of pluripotency markers (e.g. OCT4) (Supplementary Figure S10A and B). ChIP-seq was performed in these cells, and replicate datasets combined before peak calling for comparison with FOXK2 binding regions in ESCs and MESE cells (Supplementary Table S5). Again we observed a high congruence of FOXK2 binding across all three cell types (45 294 representing >81% of FOXK2 peaks in ESCs) (Figure 4A; Supplementary Figure S11A; Supplementary Table S6). However, we also observed FOXK2 binding regions that are shared by two cell types or unique to a single cell type. We identified 15 948 regions that are unique to NPCs (defined as NPC-specific). The distribution of these peaks throughout the genome differed from the genomic average but was more focussed in promoter-distal regions rather than around the TSS as observed for all FOXK2 binding events (compare Figure 4B; 8% at TSS with Figure 1B; 23% at TSS). Consistent with a potential role in neuronal-specific gene regulation, the NPC-specific FOXK2 binding regions are associated with genes corresponding to GO terms covering biological processes such as oligodendrocyte differentiation and glial cell fate commitment (Figure 4C). Two of the highest ranking motifs identified in the NPC-specific FOXK2 binding regions are for LHX2 and SOX transcription factors (Figure 4D), both known regulators of neuronal differentiation (46,47). NPC-specific FOXK2 binding events are associated with active chromatin marks that increase in NPCs (Figure 4E and F; Supplementary Figure S11B) as exemplified by the *MPPED1* locus (Figure 4G). *De novo* FOXK2 binding in NPCs is therefore associated with increased levels of activating chromatin mark deposition and neuronal specific events, consistent with a potential role in contributing to the activation of gene regulatory events in this lineage.

### FOXK2 co-binds with different transcription factors in different lineages

**Figure 5.**
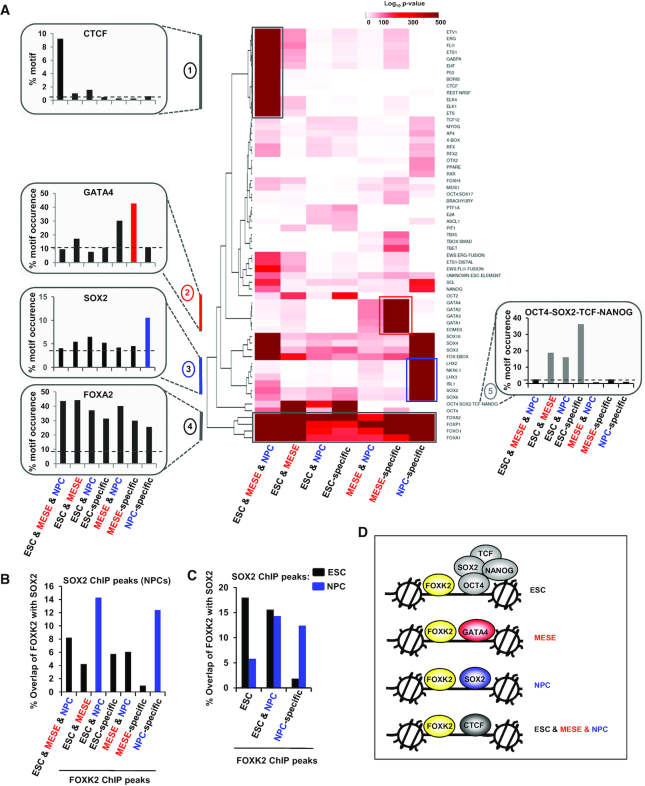
Differential TF motif enrichment associated with FOXK2 binding regions in different cell types. (**A**) Heatmap showing the enrichments of DNA binding motifs corresponding to the indicated transcription factors across each of the subcategories of FOXK2 binding peaks. The top 20% of peaks are used in each case. Data are shown according to the scale bar (log_10_*P*-value). Motifs that are specifically enriched in a particular peak category are highlighted and the percentage of FOXK2 peaks in each peak category containing each of the indicated representative motifs (from clusters 1–5) is shown on the left and right of the heatmap. Dotted line is the average background signal. (**B**) Overlap of SOX2 ChIP-seq binding peaks in NPCs (48) with FOXK2 peaks from each of the indicated subcategories of FOXK2 binding regions (data are shown as percentage of FOXK2 peaks in a given category). (**C**) As in (B) but shows the overlap of SOX2 ChIP-seq binding peaks in ESCs (black bars) or NPCs (blue bars) with FOXK2 peaks from each of the indicated subcategories of FOXK2 binding regions. (**D**) Summary model showing examples of the predominant co-bound partners for FOXK2 in the indicated different cell types.

The availability of FOXK2 binding datasets in three different cell types enabled us to further investigate the characteristics of different types of FOXK2 binding regions, and in particular the fate of the FOXK2 binding regions identified in ESCs. We initially focussed on the DNA binding motifs discovered in each subcategory of FOXK2 binding region. While all categories of peaks showed enrichment of FOX motifs as expected, they differed in the other enriched motifs. For example, the ESC-specific peaks contained high levels of OCT4 motifs (Supplementary Figure S11C). The ESC&MESE shared and ESC&NPC shared regions were similarly enriched in OCT4 binding motifs, but also showed differential enrichment of other motifs for ETS and SOX transcription factors respectively (Supplementary Figure S11C). We further examined the repertoire of binding motifs across all of the FOXK2 binding region categories and found striking enrichment of motifs in one or more categories. For example, motifs for EOMES and GATA transcription factors are most enriched in MESE-specific regions whereas motifs for SOX and LHX2 transcription factors are most enriched in NPC-specific regions (Figure 5A; Supplementary Figure S11D). Interestingly, CTCF motifs are most enriched in the regions bound in all cell types, suggesting a potential role for FOXK2 in maintaining genomic structural integrity across different cell types. Conversely, the OCT4–SOX2–TCF–NANOG composite motif is enriched in regions bound exclusively in ESCs or in ESCs plus one other cell type but is virtually absent in regions that are specific to NPCs or MESE cells. Thus, we have two scenarios, where either novel DNA binding motifs are revealed in a cell-type specific manner, or other motifs are retained alongside FOXK2 binding in the transition from ESCs to either NPCs or MESE cells. This latter finding is particularly intriguing as it suggests a priming role for FOXK2 and its associated factors in ESCs that is retained in these later lineages.

As SOX motifs are enriched in FOXK2 occupied sites in NPCs, we investigated potential FOXK2 and SOX2 co-occupancy by comparing our data to SOX2 ChIP-seq data in NPCs (48). We overlapped the SOX2 binding regions with the different categories of FOXK2 binding peaks and found the strongest overlap in the NPC-specific peaks and the peaks retained in NPCs from ESCs (ESC&NPC shared category) (Figure 5B). SOX2 plays a role in both ESC pluripotency and NPC differentiation (reviewed in 49), therefore we also compared overlaps between SOX2 binding peaks found in ESCs with FOXK2 binding events. In contrast to the large overlap seen between SOX2 binding in NPCs and FOXK2 in the NPC-specific FOXK2 binding regions, a lower degree of overlap was observed with SOX2 binding in ESCs and FOXK2 binding in NPC-specific regions (Figure 5C). Conversely, when examining regions bound by FOXK2 in both ESCs and NPCs, we found high levels of SOX2 binding in both cases. Thus binding of FOXK2 in ESCs often occurs with concomitant binding of SOX2 and is retained in NPCs, and would be consistent with one or both factors potentially priming regulatory regions at an early stage. Previously, NANOG has been shown to cooperate with SIN3A to activate pluripotency genes (50). As the NANOG motif is common in ESC-specific regions, we examined the overlap of FOXK2 and NANOG in ESCs (51) and explored co-occupancy with SIN3A. We found that 4029 FOXK2 peaks overlapped with NANOG and of these, 1226 peaks also co-bound SIN3A. Consistent with a potential role in activation, triply bound regions are associated with genes expressed at a higher level than when FOXK2 is bound alone or in combination with NANOG (Supplementary Figure S11E). Similarly the regions co-bound by FOXK2, NANOG and SIN3A contain significantly higher levels of histone marks typically associated with gene activation (Supplementary Figure S11F). These results therefore build on the developing theme of FOXK2 being associated with transcriptional activating events.

Collectively, these results lead to a model in which there is lineage-specific binding of FOXK2 alongside transcription factors that play important lineage-specific roles (Figure 5D). Importantly, there are also other regions which are bound by FOXK2 in both ESCs and NPCs and there, FOXK2 tends not to be associated with lineage-specific transcription factors but instead is associated with ESC-specific transcription factors, or other transcription factors like SOX2 which are re-purposed as differentiation proceeds.

### FOXK2 associates with regions that are active in NPCs but primed in ESCs

Our overlapping ChIP-seq datasets enabled us to identify regions that are occupied by FOXK2 in ESCs and where binding is maintained either in the MESE cells or NPCs. Such behaviour suggests either that the regions may be already active in ESCs and/or primed for activation either in MESE cells or NPCs or further more differentiated cell lineages that emerge later. We focussed on FOXK2 binding regions that are initially occupied in ESCs and retained in NPCs. These FOXK2 binding events (2231) showed a similar genome-wide distribution to NPC-specific FOXK2 peaks (Supplementary Figure S11G) and like the NPC-specific peaks, are associated with genes corresponding to GO terms covering biological processes related to neuronal development such as glial cell and neuron cell fate commitment (Supplementary Figure S11H).

**Figure 6.**
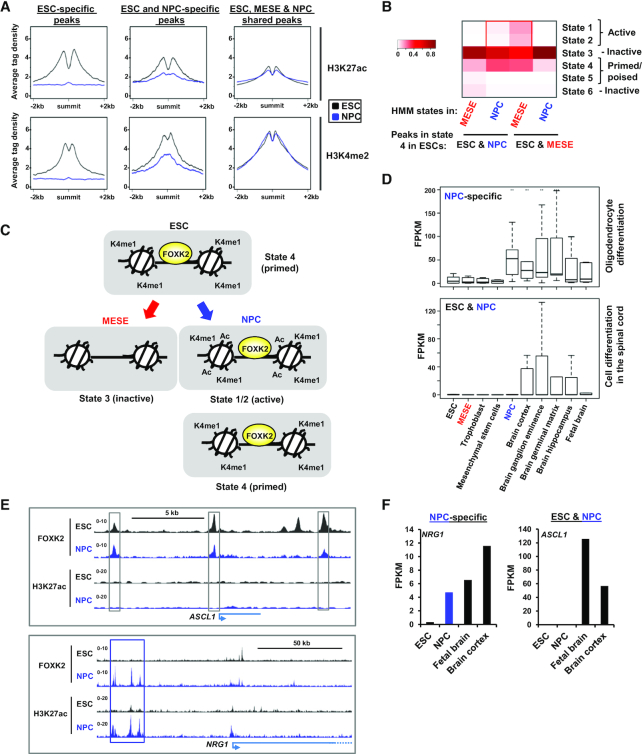
Regulatory dynamics in FOXK2 binding regions. (**A**) Average tag density plots of H3K27ac (top) or H3K4me2 (bottom) surrounding the summits (±2 kb) of the indicated categories of FOXK2 binding peaks. Data are shown for histone marks from ESCs (black) or NPCs (blue) for each category of FOXK2 peaks. (**B**) Percentage of FOXK2 peaks in HMM states in NPCs or MESE cells for the indicated classes of FOXK2-bound regions (ESC&NPC or ESC&MESE) that exist in state 4 regions (ie ‘primed enhancers’) in ESCs. (**C**) Model illustrating the chromatin state changes associated with ESC&NPC FOXK2 binding regions from ESCs to NPCs or MESE cells. (**D**) Boxplots of the expression of the FOXK2 target genes contained in the indicated GO term categories (right) across the indicated cell types/tissues. Genes associated with either NPC-specific FOXK2 binding regions (top) or ESC&NPC FOXK2 binding regions (bottom) were analysed. (**E**) UCSC genome browser views of FOXK2 and H3K27ac ChIP-seq binding profiles in ESCs and NPCs for examples of ESC&NPC FOXK2 binding (*ASCL1*; top) or NPC-specific FOXK2 binding (*NRG1*; bottom). Cell type-specific (blue) and shared (grey) regions bound by FOXK2 are boxed. (**F**) Examples of the expression of genes associated with NPC-specific (*NRG1*; left) or ESC&NPC FOXK2 binding regions (*ASCL1*; right) are shown.

To begin to understand the regulatory dynamics of the different categories of FOXK2-associated regions, we first examined the histone marks surrounding the FOXK2 peaks in ESCs and NPCs. The ESC-specific FOXK2 binding regions exhibit high levels of the activation-associated histone marks H3K27ac and H3K4me2 which are subsequently extinguished in NPCs (Figure 6A, left). In contrast the FOXK2-bound regions occupied in both ESCs and NPCs showed high levels of the two histone marks in ESCs that are partially retained in NPCs (Figure 6A, middle) suggesting that ‘retained’ FOXK2 binding correlates with retention of locally active chromatin. Indeed, this is even more apparent in the FOXK2-bound regions commonly occupied in ESCs, NPCs and MESE cells (ESC, MESE & NPC shared) which show high levels of these histone marks in both cell types (Figure 6A, right). Next we looked in more detail at more complex chromatin states defined by our HMM approach in ESCs and asked how these change in NPCs and MESE cells. We focussed on regulatory regions that are bound by FOXK2 in ESCs and NPCs, and are associated with a ‘primed’ state (i.e. high H3K4me1). We found that these regions are often either retained in this state or converted into active states (i.e. high H3K27ac) in NPCs but show reduced levels of all of these states in MESE cells (Figure 6B). Conversely, FOXK2-bound regions found in ESCs and MESE cells, show either retention of a poised state or conversion to an active state in MESE cells but not in NPCs. Thus, in both cases, retention of FOXK2 binding in a particular differentiated lineage correlates with retained or increased activity of the genomic region in that lineage (summarised in Figure 6C). This finding was further substantiated by examining ‘poised’ regions (ie high H3K4me1 and H3K27me3), as these again showed preferential conversion to more active chromatin states only in the cell types where FOXK2 binding is retained (Supplementary Figure S12A and B).

The changes seen in histone marks suggest that we should also observe changes in target gene expression, coincidental with FOXK2 binding activities. Namely we predict genes associated with FOXK2 regions that are bound specifically by FOXK2 in NPCs or already bound in ESCs and retained in NPCs will specifically be expressed in NPCs and/or later lineages. We tested this by analysing the expression of genes found in the GO categories associated with FOXK2 binding events (Figure 4D, Supplementary 11D). For NPC-specific FOXK2 binding events, we found that associated genes are inactive in ESCs but became activated in NPCs and retained their activity in several brain tissues (Figure 6D, top; Supplementary Figure S12C). Similarly, genes associated with regions bound by FOXK2 in ESCs and NPCs exhibit little activity in ESCs but become active in a range of brain tissues but not in NPCs (Figure 6D, bottom; Supplementary Figure S12C). This suggests a model whereby many ‘ESC&NPC shared’ FOXK2 bound regions and their associated genes remain in an inactive state in NPCs. Indeed the FOXK2 binding regions associated with *ASCL1* and *OLIG1* remain devoid of H3K27ac in NPCs (Figure 6E, top; Supplementary Figure S12D), which correlates with the lack of transcription in these cells (Figure 6F, Supplementary Figure S12F). This clearly differs from NPC-specific binding regions where *de novo* FOXK2 binding is generally associated with the acquisition of both activating marks (Figure 6E, bottom; Supplementary Figure S12E; Figure 4F), and the activation of target genes such as *NRG1* and *PAX6* (Figure 6F, Supplementary Figure S12G) in NPCs.

Together these results suggest a model whereby FOXK2 binding in ESCs can be retained in NPCs, and in that situation, the binding regions and target genes are generally primed for activation at later stages in neuronal differentiation. In contrast, *de novo* FOXK2 binding is associated with more immediate binding and associated target gene activation in a lineage specific manner.

### FOXK transcription factors regulate gene expression to control NPC differentiation

**Figure 7.**
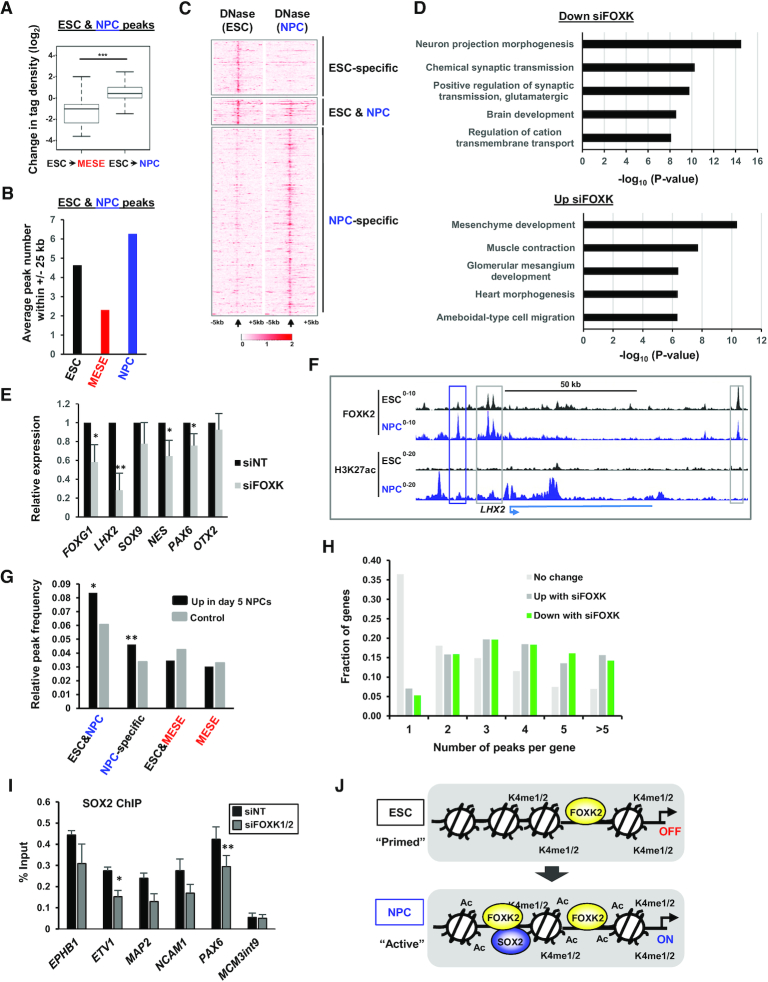
FOXK2 controls gene expression during NPC differentiation. (**A**) Fold changes in tag density of ESC&NPC FOXK2 peaks as ESCs transition to MESE or NPCs. (**B**) Average number of additional FOXK2 peaks surrounding ESC&NPC FOXK2 peaks in the indicated cell types. (**C**) Heatmaps of DNase-seq signal from ESCs (left) or NPCs (right) surrounding the summits of the ESC-specific, ESC&NPC or the NPC-specific FOXK2 binding peaks. (**D**) Top 5 GO terms associated with genes down (top) or up (bottom) regulated after 5 days differentiation to NPCs following depletion of FOXK1/2. (**E**) RT-qPCR analysis of the expression of the indicated genes following depletion of FOXK1/2 relative to a non-targeting control (NT, taken as 1) after 5 days differentiation to NPCs. (**F**) UCSC genome browser views of FOXK2 and H3K27ac ChIP-seq binding profiles in ESCs and NPCs at the *LHX2* locus. NPC-specific (blue) and ESC&NPC (grey) FOXK2 binding peaks are boxed. (**G**) Relative peak frequency of the indicated categories of FOXK2 bound sites associated within the region −10 kb to +1 kb of the TSS of genes that are both upregulated at day 5 of differentiation to NPCs and downregulated following FOXK1/2 depletion. Controls are gene lists that are not affected by FOXK2 depletion. Asterisks refer to *P*-values; * = <0.05, ** = <0.01 relative to expected frequency across all genes associated with the peak type. (**H**) Fraction of genes in the indicated datasets (up/down in NPCs with FOXK1/2 depletion or unchanged) containing the indicated numbers of peaks (defined as peaks within 10 kb of either direction from the TSS). (**I**) ChIP analysis of SOX2 binding to FOXK2 bound regions associated with the indicated genes following FOXK1/2 depletion or non-targeting (NT) siRNA treatment during NPC differentiation for 5 days. Data are shown as SEM (n = 3). Asterisks refer to *P*-values; * = <0.05, ** = <0.01. (**J**) Model showing the premarking by FOXK2 in ESCs of genes which become activated in NPCs which acquire additional NPC-specific FOXK2 binding regions, associated with lineage-specific TF motifs.

To further explore the role of FOXK2 during NPC differentiation, we extended our investigation of the properties of the FOXK2 bound regions that are present in ESCs and NPCs. These peaks show increased FOXK2 binding in the transition to NPCs but a decrease during differentiation to MESE cells (Figure 7A). Moreover, there is an increase in the number of additional FOXK2 peaks found surrounding these peaks in NPCs and decrease in MESE cells (Figure 7B), as exemplified by the *PAX6* and *LHX2* loci (Figure 7F; Supplementary Figure S12E). This indicates a more complex regulatory environment is developed surrounding the ‘sentinel’ FOXK2 peaks found in ESCs and retained in NPCs as the cells differentiate. However, these sentinel peaks are already found in regions of open chromatin in ESCs whereas the NPC-specific peaks show a high degree of chromatin opening as differentiation proceeds (Figure 7C; Supplementary Figure S13).

Next we directly assessed whether FOXK2 impacts on gene expression control during stem cell differentiation. FOXK1 is a closely related paralogue of FOXK2 and both increase in expression during the transition to NPCs (see Supplementary Figure S14E) and have the potential to functionally compensate for the loss of other. We therefore depleted both FOXK2 and FOXK1 in ESCs and assessed the gene expression changes associated with differentiation towards NPCs (Supplementary Tables S7 and S8). First we assessed the impact of FOXK depletion in ESCs (Supplementary Figure S14A, left panel) and found roughly equal numbers of genes were up and downregulated following FOXK depletion (Supplementary Figure S14B). Next, we compared this with the depletion of its coregulatory partner SIN3A (Supplementary Table S7; Supplementary Figure S14A, right panel). Again we observed similar numbers of genes that were up or downregulated following SIN3A depletion (Supplementary Figure S14B). Importantly, there was a significant overlap in their targets in both the up- and down-regulated categories. Genes which were upregulated following depletion of FOXK or SIN3A are associated with autophagy (Supplementary Figure S14C) which is consistent with previous conclusions on the repressive roles of these transcriptional regulators (14). However, equally these results are not consistent with a sole repressive role but suggest a role in transcriptional activation, as also suggested by the association of FOXK2 and active regulatory regions in the genome.

To further explore the activating role of FOXK transcription factors we depleted FOXK in ESCs (Supplementary Figure S14D and E) and examined gene expression following 5 days of differentiation towards NPCs. Again we observed a roughly equal split of genes going up and down (604 up and 598 down; averaged TP10M > 0.1 and median of fold change of three replicates > 1.5) (Supplementary Table S8) with those downregulated being associated with various neuronal-associated GO terms (Figure 7D). Indeed, several neuronal marker genes including those encoding the transcription factors, FOXG1, PAX6 and LHX2 show reduced expression following FOXK depletion (Figure 7E; supplementary Figure S14E). The *LHX2* locus contains several sentinel FOXK2 peaks that are established in ESCs and retained in ESCs and an additional peak that appears in NPCs (Figure 7F). More generally, we examined FOXK2 binding close to genes whose expression increases in the transition between ESCs and NPCs and are downregulated following FOXK depletion. We found that these genes are more likely associated with NPC-specific and/or ESC & NPC shared FOXK2 peaks (Figure 7G) indicating a direct connection between FOXK2 binding and their regulation. Furthermore, we also observed that genes regulated by FOXK in NPCs showed a higher number of binding events than unregulated genes, with most being associated with three or more FOXK2 peaks (Figure 7H). A similar phenomenon was observed for multiple FOXK2 binding events associated with FOXK1/2-regulated genes in ESCs (Supplementary Figure S14F). To examine the consequences of FOXK2 binding, we asked whether FOXK2 is required for the redistribution of SOX2 that is observed in NPCs and found that FOXK depletion reduced both FOXK2 (Supplementary Figure S14G and H) and SOX2 occupancy (Figure 7I) at co-bound regions in NPCs. Phenotypically, NPC differentiation was perturbed as exemplified not only by the reduction in expression of NPC-specific transcription factors but also a reduction in NPCs exhibiting expression of the neural related cell surface markers CD15 (SSEA1) and CD56 (NCAM1) (Supplementary Figure S14I and J).

Collectively, these data demonstrate that FOXK transcription factors contribute to NPC differentiation. Importantly, they contribute to both transcriptional repression and transcriptional activation with the latter contributing to activation of NPC-specific gene expression and is consistent with the connections between FOXK2 binding and areas of active chromatin.

## DISCUSSION

Each type of human cell contains a unique portfolio of enhancers and promoter proximal regulatory regions which control gene transcription. These are dynamically formed and decommissioned throughout the course of development. Due to their ability to access and bind to DNA packaged into chromatin, the FOXA transcription factors have been established as paradigms for pioneers which can begin the enhancer activation process, and do so in a cell type-specific manner (2). More recently, the related FOXD3 and FOXH1 transcription factors have been shown to play a role in enhancer priming during differentiation (3,4). Both of these proteins recruit repressive factors which keep the regulatory element silent prior to the recruitment and handover to lineage-specific transcription factors that lead to enhancer activation. Here, we investigate another FOX transcription factor, FOXK2, and demonstrate that it is also recruited to enhancer regions prior to their activation but differs in that binding is retained as enhancers become activated during differentiation (see Figure 7J), indicating a potential role in both priming and maintaining enhancer activity.

FOXK2 binding to regulatory regions is extensive in ESCs, and there is a striking retention of many of these binding sites in other cell types. This is in keeping with the ubiquitous expression of FOXK2 and its close paralogue FOXK1. FOXK2 binding is generally associated with marks of active chromatin. However as new active regulatory regions emerge during differentiation, new FOXK2 binding events emerge which might reflect pioneering activity. However, more interestingly, many regions that are prebound by FOXK2 in ESCs retain FOXK2 binding but gain active histone marks. Thus FOXK2 marks genomic regions for future activation at an early stage in the differentiation process. Again, it is possible that FOXK2 acts as an early pioneer factor at a cell stage that precedes that in the H1 ESCs examined here. Through focussing on differentiation to the NPC lineage we demonstrate the relationship of the newly activated regions for gene activation specifically in NPCs and subsequent neuronal lineages. These observations are incompatible with a simple model in which FOXK2 acts as a transcriptional repressor to shut down gene transcription as implied by multiple studies that have linked FOXK1 and FOXK2 to co-repressor complex binding (6–13). Indeed, we demonstrate that depletion of the FOXK transcription factors leads to both increases and decreases in gene expression, which indicates a role for FOXK transcription factors in balancing the transcriptional output of a given gene.

A role in transcriptional activation is also implied by the tight association between FOXK2 and regions harbouring active chromatin marks such as H3K27 acetylation. Surprisingly, we also found that its binding partner, the corepressor SIN3A and associated histone deacetylase HDAC2 is also found at regions of active chromatin. This suggests a model where the FOXK2 and the SIN3A-HDAC complex balances the level of histone acetylation rather than completely removing it in this scenario. Indeed, the SIN3A complex has been implicated as both a corepressor and also as a potential coactivator for Nanog in promoting ESC pluripotency (13). SIN3A has also been shown to be required for full gene activation in the hypoxic response (52). Earlier studies in yeast suggested a role for SIN3 in both repression and activation (53) and a more recent study demonstrated activator-mediated recruitment of SIN3 (54). It is not clear how SIN3 adopts different activities at different FOXK2 associated sites. However, the recent finding that SIN3A and SIN3B associate with a variety of different proteins opens up the possibility that different co-factors may be recruited at different sites (55). Indeed in yeast, SIN3 is associated with two different complexes, one of which suppresses intragenic transcription and hence contributes to the canonical gene activation process (56). More generally, genome-wide mapping of a range of different HDACs demonstrated a strong correlation with active transcription, and more surprisingly areas of high histone acetylation (57). Our results build on these findings and suggest a more nuanced view of transcription repressor and corepressor proteins that balance activation levels rather than acting as a unidirectional switch.

As cells differentiate to new lineages such as NPCs, retained or *de novo* FOXK2 binding becomes associated with regions containing binding motifs for transcription factors that are relevant for that cell type. The transcription factor LHX2 is one such example, and LHX2 is expressed specifically in NPCs. However we also observed genome-wide repositioning of SOX2 binding during the transition from ESCs to NPCs as new regulatory regions bound by FOXK2 emerge, while SOX2 binding is retained at sites where FOXK2 binding is also retained. Similarly, FOXK2 binding is associated with a distinct set of transcription factors during differentiation to MESE cells. Thus no combinatorial code exists and rather FOXK2 marks regions that can be bound by the dominant transcriptional regulators in a given cell type. It is possible that there may also be an interplay with other lineage-specific FOX transcription factors as we recently demonstrated that genomic binding of FOXK2 is shared with other FOX transcription factors, leading to a tread milling model for partial occupancy by any given FOX protein at the same time (58). Further work is needed to establish whether such lineage-specific FOX proteins contribute to a balanced regulatory activity at these regions.

In summary, our work provides an extensive analysis of FOXK2 binding in ESCs and downstream linages, and leads to the finding that FOXK2 binding premarks regulatory regions for future activation. More generally, FOXK2 binding is associated with active chromatin regions, which is consistent with a role in transcriptional activation in addition to its well established role as a transcriptional repressor protein.

## DATA AVAILABILITY

Our ChIP-seq and RNA-seq data have been deposited with ArrayExpress. Accession numbers: FOXK2 ChIP-seq in ESCs, MESE cells and NPCs ( E-MTAB-9630). FOXK1/K2 and SIN3A siRNA RNA-seq experiments in ESCs and FOXK1/2 siRNA experiments in differentiating NPCs (E-MTAB-9639).

## Supplementary Material

gkaa1281_Supplemental_Files
