# Correction to article “The forkhead transcription factor FOXK2 premarks lineage-specific genes in human embryonic stem cells for activation during differentiation’’

**DOI:** 10.1093/nar/gkab221

**Published:** 2021-03-22

**Authors:** Zongling Ji, Yaoyong Li, Sean X Liu, Andrew D Sharrocks

**Affiliations:** Faculty of Biology, Medicine and Health, University of Manchester, Michael Smith Building, Oxford Road, Manchester M13 9PT, UK; Faculty of Biology, Medicine and Health, University of Manchester, Michael Smith Building, Oxford Road, Manchester M13 9PT, UK; Faculty of Biology, Medicine and Health, University of Manchester, Michael Smith Building, Oxford Road, Manchester M13 9PT, UK; Faculty of Biology, Medicine and Health, University of Manchester, Michael Smith Building, Oxford Road, Manchester M13 9PT, UK

The authors wish to correct the Funding statement of their article (1) to:

## FUNDING

Wellcome Trust; BBSRC. Funding for open access charge: Institutional award from Wellcome (103857/Z/14/Z) and RCUK (BB/R002851/1).

This change does not affect the results, discussion and conclusions presented in the article. The published article has been updated.
