# Supplementary material for: The forkhead transcription factor FOXK2 premarks lineage-specific genes in human embryonic stem cells for activation during differentiation
Source: Nucleic Acids Res. 2021 Jan 12;49(3):1345–63. doi: 10.1093/nar/gkaa1281 (PMC7897486; doi:10.1093/nar/gkaa1281)
Supplement: gkaa1281_Supplemental_Files [file gkaa1281_supplemental_files.zip › Supplementary Figures&legends.pdf]

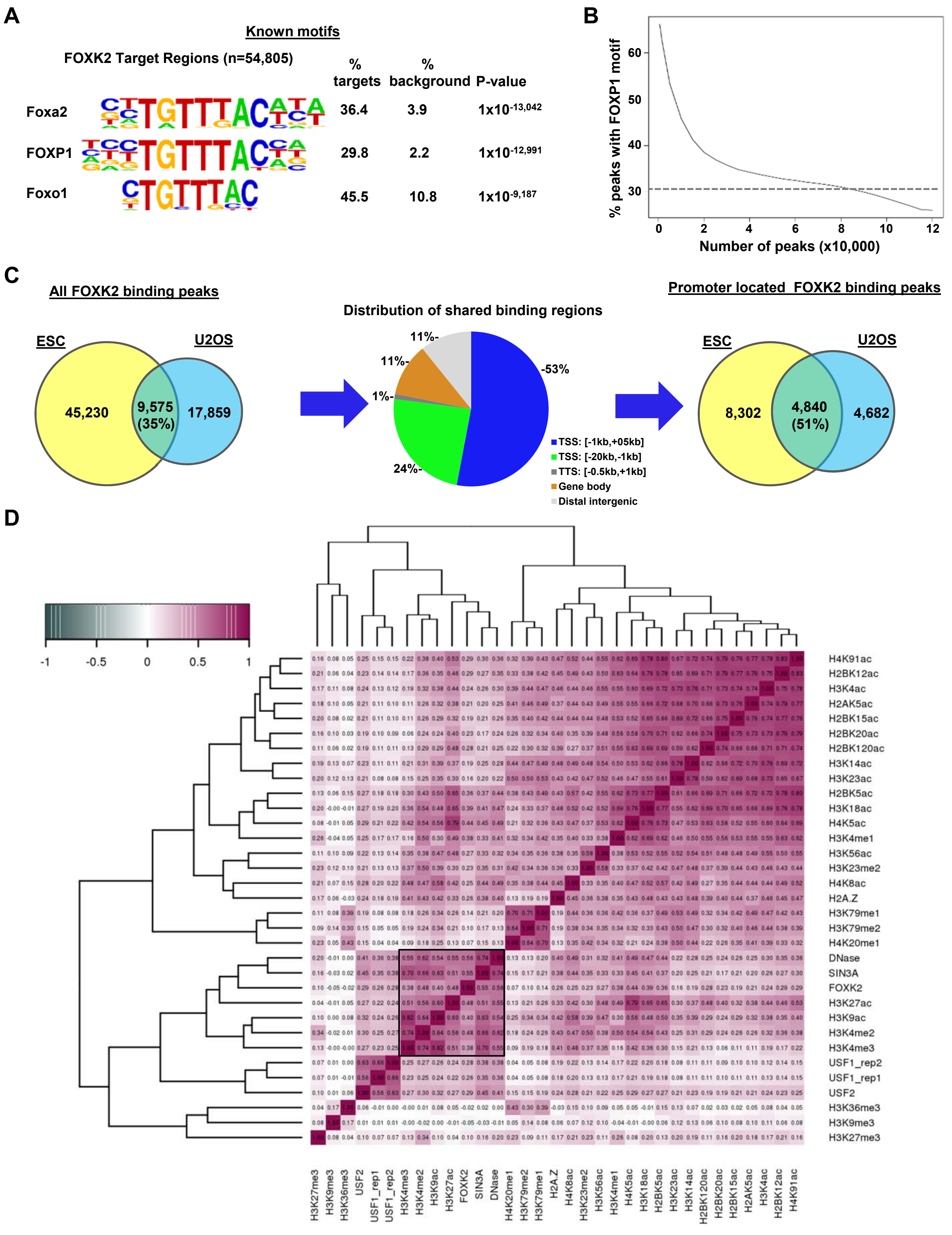

Supplementary Figure S1. FOXK2 ChIP-seq in human ESCs. (A) Known motif discovery in FOXK2 binding regions. The top three motifs are shown and all represent motifs from previous FOX family ChIP-seq datasets. (B) Frequency of FOXP1 motifs found in FOXK2 ChIP-seq data following peak splitting. Peaks are ranked by q-value in sliding bins of 5000 peaks. (C) Venn diagrams showing the overlap of the FOXK2 binding regions identified in human H1 ESCs and U2OS cells (52; E-MTAB-2204) for all binding regions (left) and promoter proximal regions (right). The distributions of all FOXK2 binding regions shared between H1 and U2OS cells across different genomic locations are shown in the middle. (D) Hierarchical clustering of Pearson's correlation coefficients between ChIP-seq data for FOXK2, SIN3A and the indicated histone modifications (magnified version of Fig. 1C).

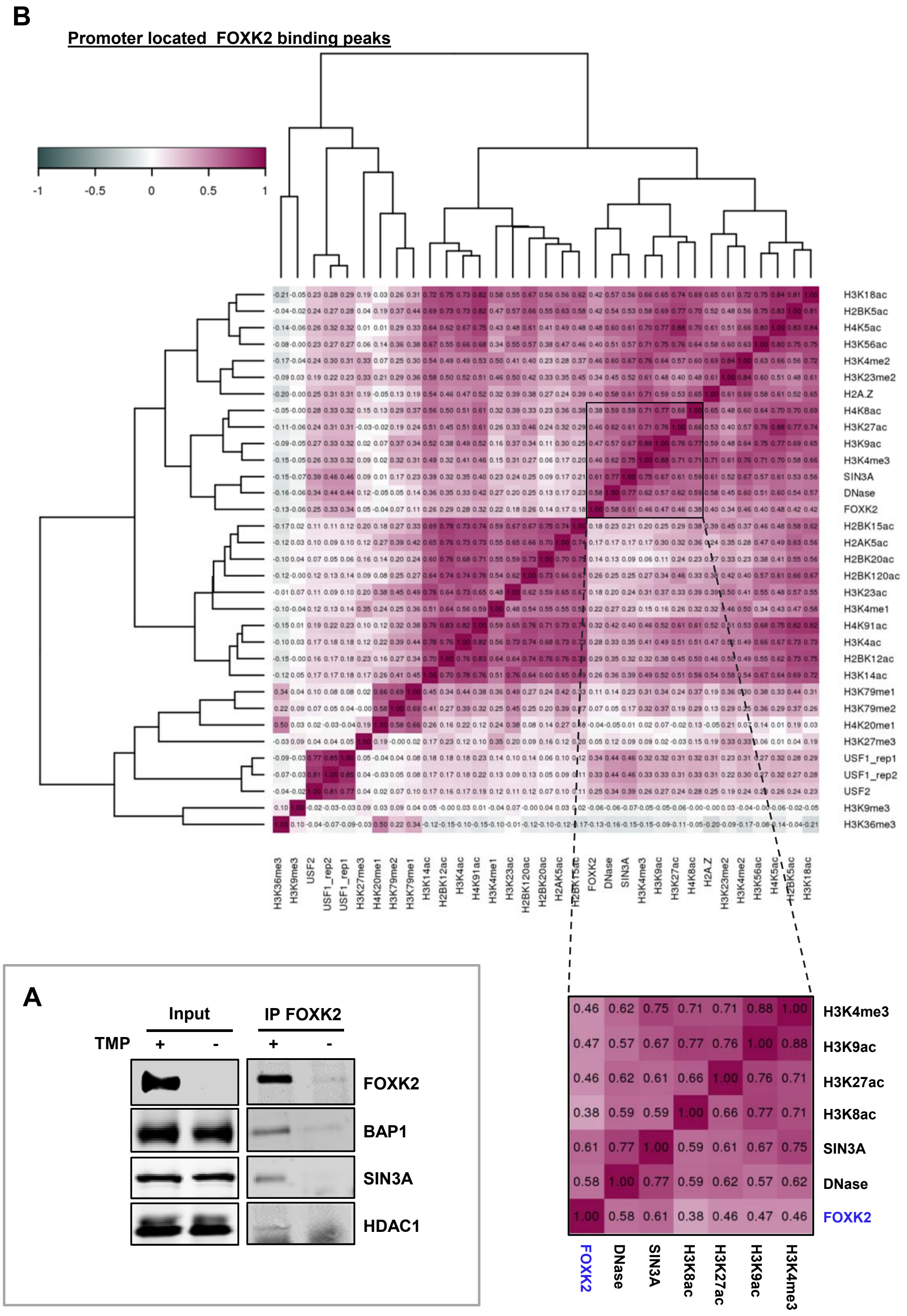

Supplementary Figure S2. Correlations between FOXK2 promoter binding and histone marks. (A) Co-immunoprecipitation (co-IP) analysis of FOXK2 with the indicated proteins in H1-FOXK2-DHFR cells harboring a TMP-sensitive FOXK2 fusion protein. The presence of TMP is indicated and input protein levels are shown on the left. (B) Hierarchical clustering of Pearson's correlation coefficients between ChIP-seq data for FOXK2, SIN3A and the indicated histone modifications for FOXK2 binding regions located close to the promoter (-1/0.5 kb to TSS). Datasets closely related to FOXK2 are highlighted.

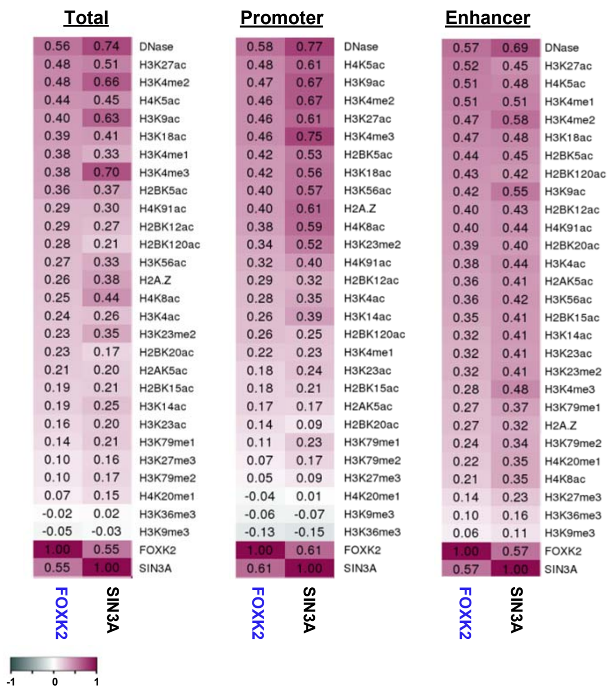

Supplementary Figure S3. Correlations between FOXK2 promoter binding and histone marks. Pearson's correlation coefficients between ChIP-seq data for FOXK2 or SIN3A and the indicated histone modifications for all FOXK2 binding regions or regions located close to the promoter (-1/+0.5 kb to TSS) or putative enhancers (ie all other regions outside of the defined promoter regions). Data are ranked according to similarity to FOXK2, and corresponding co-associations with SIN3A are also shown.

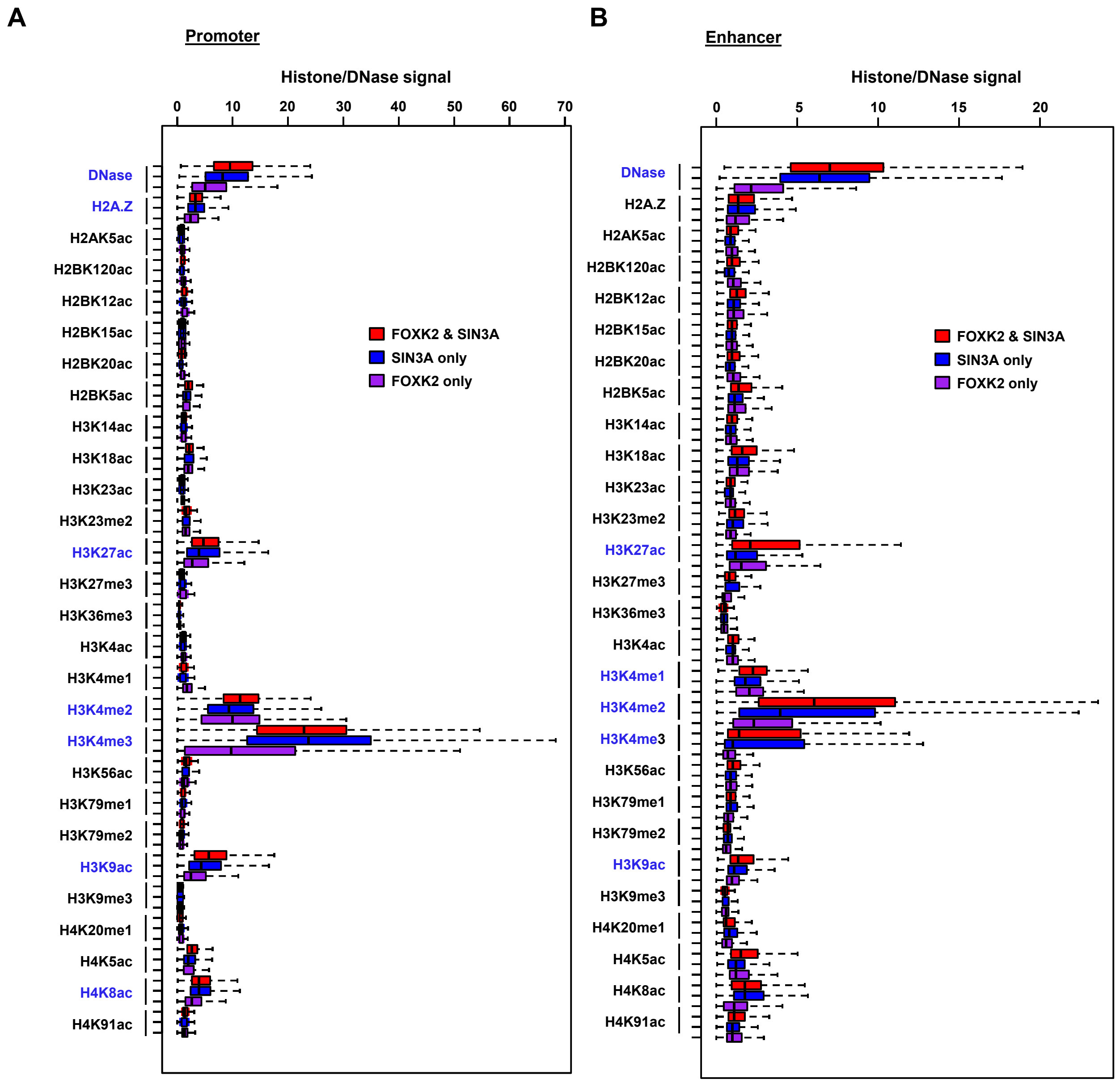

Supplementary Figure S4. Histone modifications associated with FOXK2 and SIN3A binding regions. (A and B) Boxplots of ChIP-seq signals for DNase sensitivity or the indicated chromatin marks at regions co-bound by FOXK2 and SIN3A or either factor alone. Data are shown for FOXK2 binding regions located close to the promoter (-1/0.5 kb to TSS)(A) or in putative enhancers (ie all other regions outside of the defined promoter regions)(B). Horizontal line represents median expression and whiskers extend to the most extreme data point which is no more than 1.5 times the interquartile range from the box. The data for promoter and enhancer regions are roughly similar with the histone marks showing greatest levels at binding regions for FOXK2 and SIN3A highlighted in blue. However, as expected, promoter regions do show high levels of H2A.Z and H3K4me3 whereas enhancers show higher levels of H3K4me1.

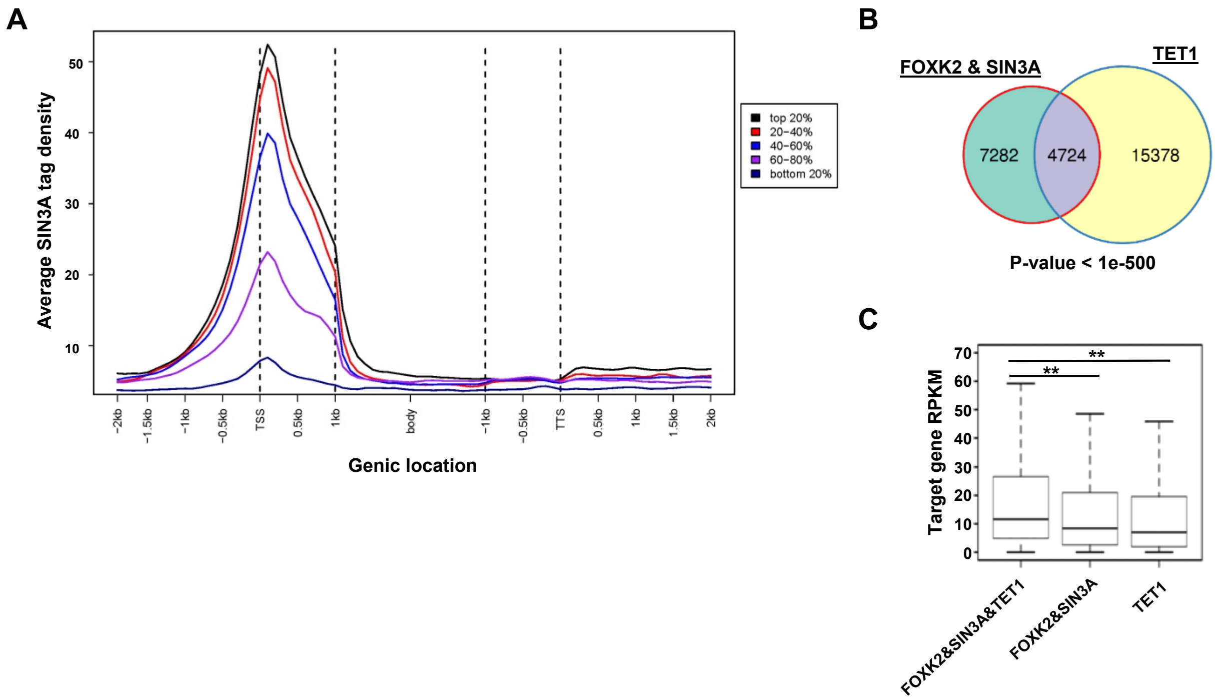

Supplementary Figure S5. SIN3A binding correlates with target gene activity levels. (A) Average tag density plot of the SIN3A ChIP-seq signal around target genes partitioned into quintiles according to their expression level in H1 ESCs. SIN3A tag counts are aggregated in 100 bp bins in 3kb regions surrounding the TSS (-2kb to +1kb) and TTS (-1kb to +2kb). (B) Venn diagram showing the overlap of regions bound by FOXK2&SIN3A or TET1 in ESCs. (C) Box plot showing the levels of target gene (defined as having the indicated peak combinations in their promoter regions; -1kb to 500bp) activity (RPKM) in ESCs. Horizontal lines on the boxes represent median expression; **= p-value<0.01.

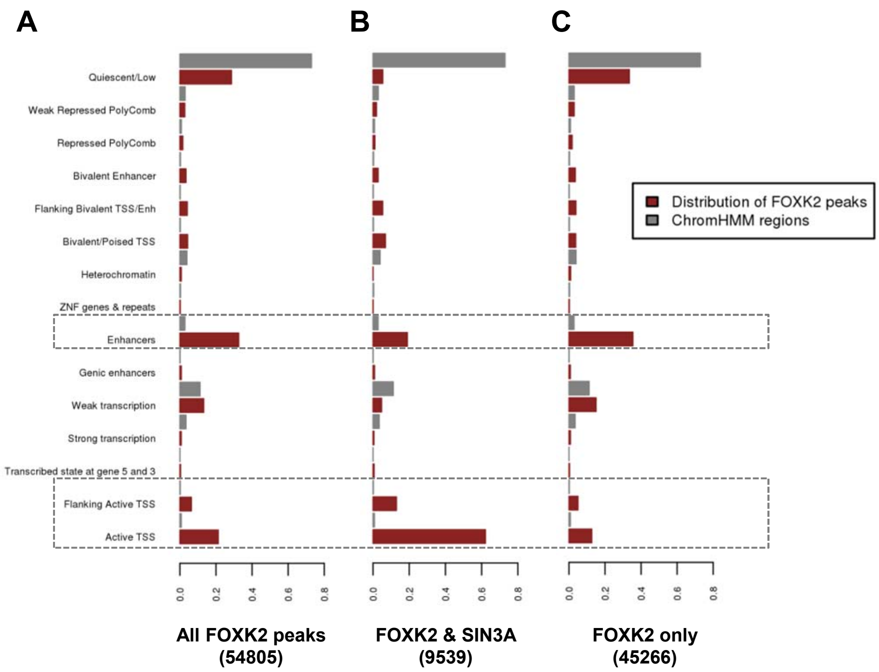

Supplementary Figure S6. Association of FOXK2 binding regions with different chromatin regions. (A-C) The distribution of FOXK2 binding regions with the indicated categories of genomic binding regions (28) is shown relative to the total distribution of these regions. Data are shown for all FOXK2 peaks (A), regions bound by FOXK2 and SIN3A (B) or by FOXK2 only (C). Regulatory regions around the TSS or intergenic enhancers are highlighted. The major difference is the stronger association with active promoters for regions co-bound by FOXK2 and SIN3A.

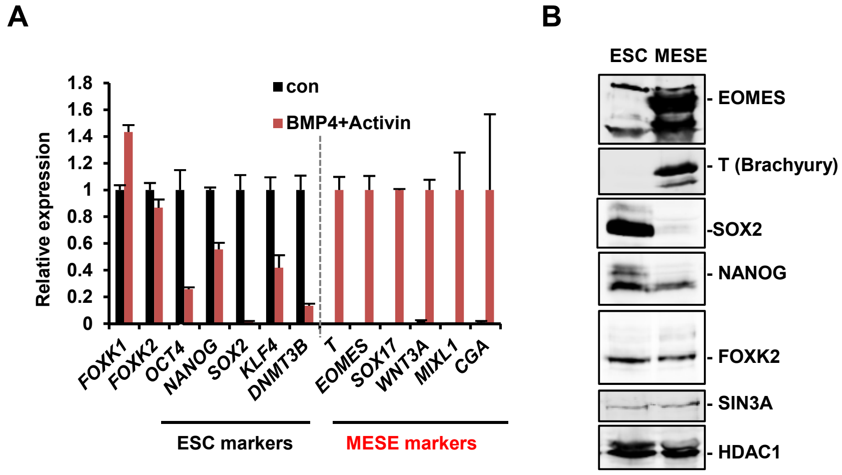

Supplementary Figure S7. Validation of mesendodermal differentiation protocol. (A) RT-qPCR analysis of the indicated genes in H1 ESCs either untreated (con) or treated with BMP4 and activin for 2 days. Data are normalized for *HMBS* expression and their levels normalized to expression in either ESCs (left) or MESE cells (right) (taken as 1) and are the average of technical replicates. (B) Western analysis of the indicated proteins in ESCs or MESE cells in the cells used for the two ChIP-seq experiments.

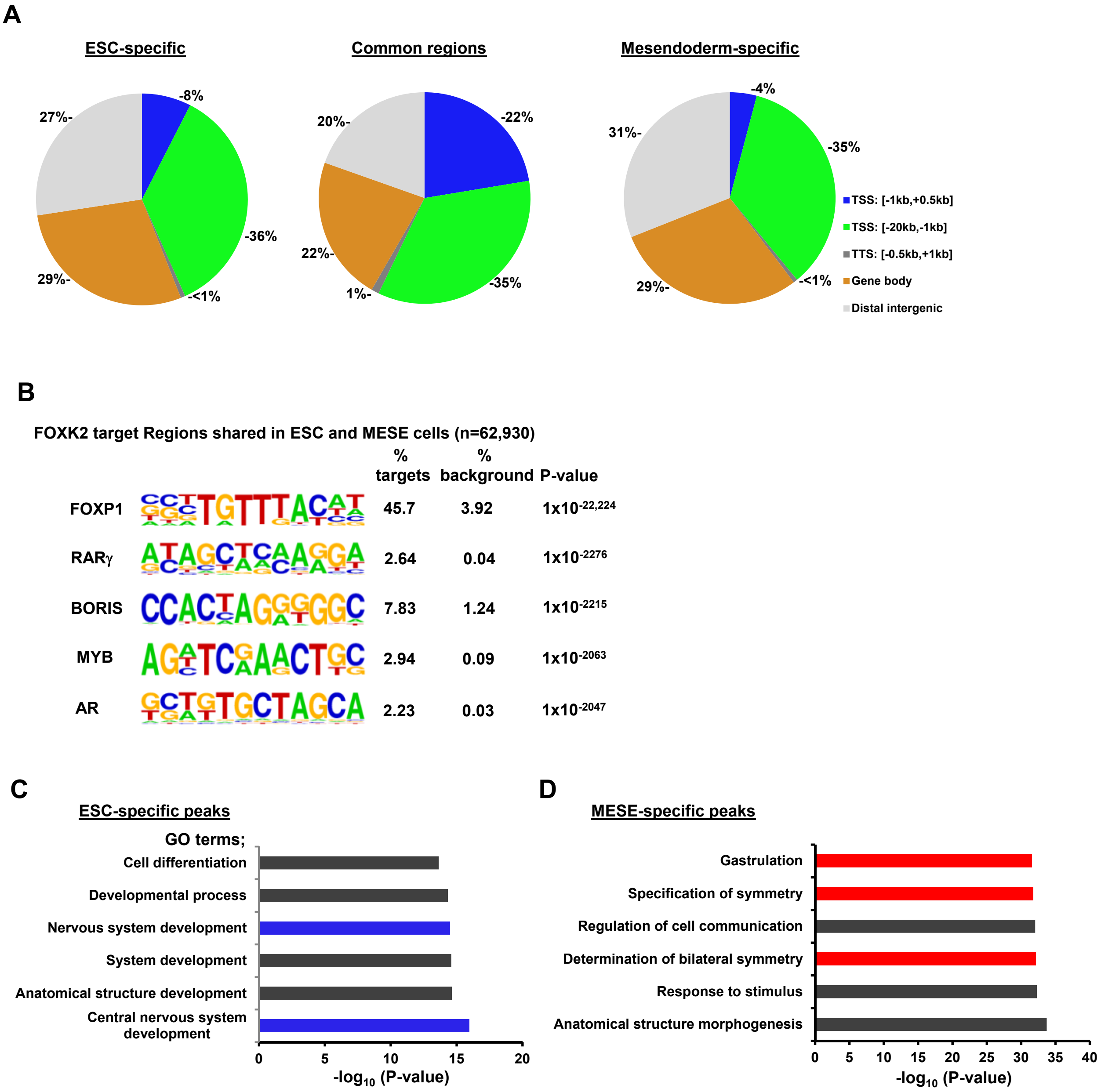

Supplementary Figure S8. Features of FOXK2 binding regions in ESCs and mesendodermal cells. (A) Distributions of FOXK2 binding regions across different genomic locations. Data are shown for ESC-specific binding regions (left), MESE-specific binding regions (right) and regions commonly bound in both cell types (centre). (B) De novo motif discovery in FOXK2 binding regions shared in ESCs and mesoendodermal cells. Note that although the probability scores are high, the percentage of regions containing the motif is low, and therefore the lack of a dominant binding partner(s). (C and D) Top GO terms for the genes associated with FOXK2 binding regions that are specifically occupied in either ESCs (C) or MESE cells (D). These terms form part of the REVIGO analysis in Fig. 2G and H.

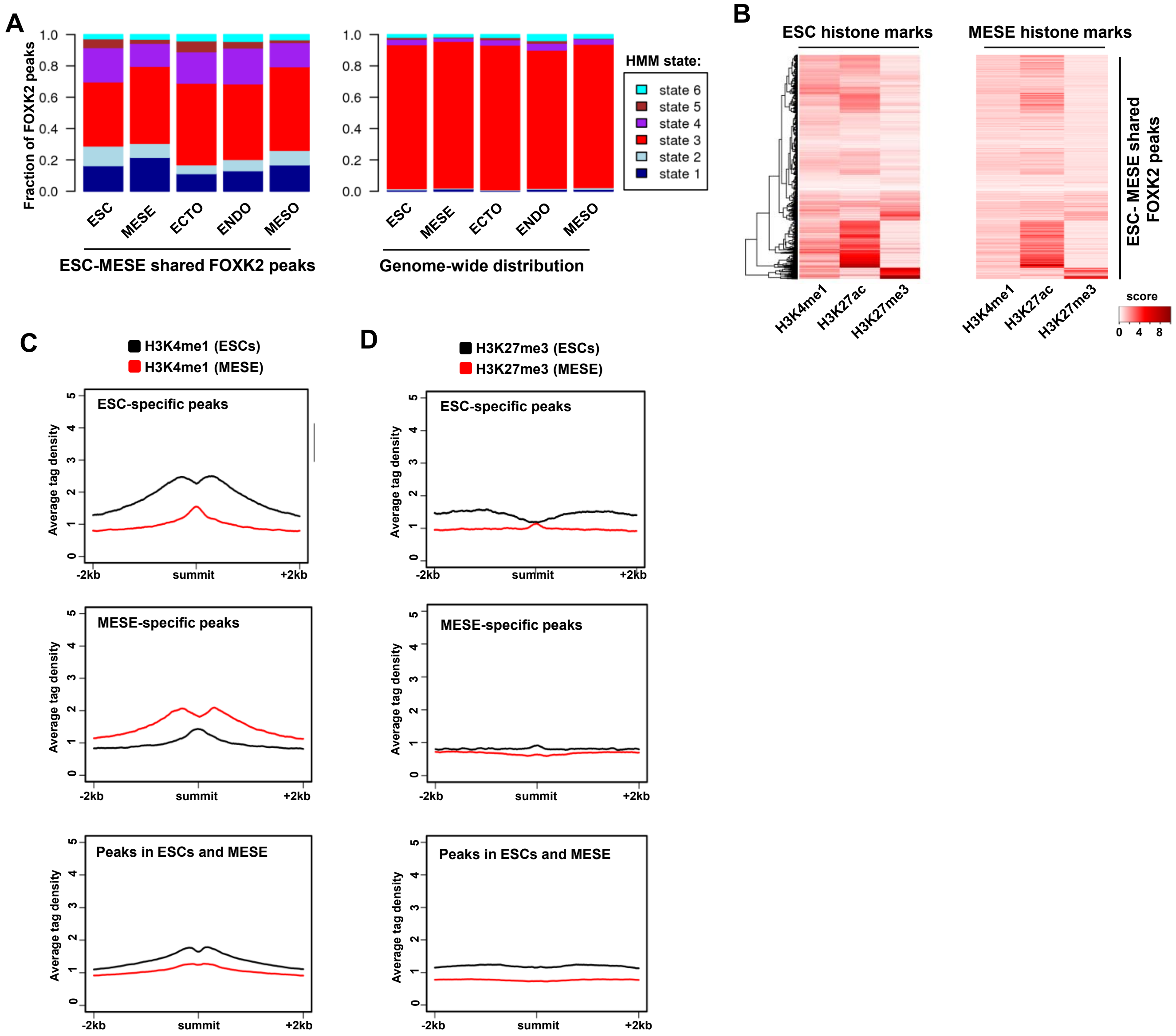

Supplementary Figure S9. Fate of ESC-specific and ESC&MESE FOXK2 peaks during differentiation. (A) HMM states for regions containing the ESCs&MESE category of FOXK2 peaks in ESCs and the indicated differentiated cell types (MESE=mesendoderm; ECTO=ectoderm; ENDO=endoderm; MESO=mesoderm). The genome-wide distribution of the states is shown on the right (see Fig. 3A for key to state names). (B) Heatmap showing the relative read density of the indicated histone modifications around the ESC&MESE category of FOXK2 peaks. Data are show for histone marks in ESCs (left) or MESE cells (right). Data are normalised numbers of reads overlapping with peaks, capped at a value of 10. (C and D) Average tag density plots of H3K4me1(C) or H3K27me3 (D) levels from either ESCs (black line) or MESE cells (red line) in a 4 kb window around the summit of the indicated categories of FOXK2 peaks.

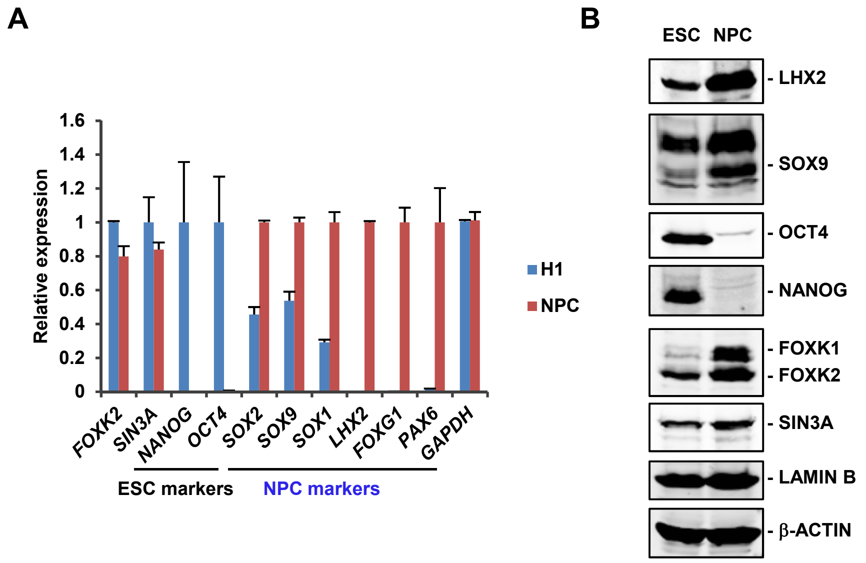

Supplementary Figure S10. Validation of NPC differentiation protocol. (A) RT-qPCR analysis of the indicated genes in H1 ESCs either untreated (blue) or treated with NPC differentiation media for 7 days (red). Data are normalized for *HMBS* expression and their levels normalized to expression in either ESCs for ESC markers (left) or NPCs for NPC markers (right) (taken as 1) and are the average of technical replicates. (B) Western analysis of the indicated proteins in cell lysates from NPCs used for one of the ChIP-seq experiments with undifferentiated H1 ESC cell lysates as control.

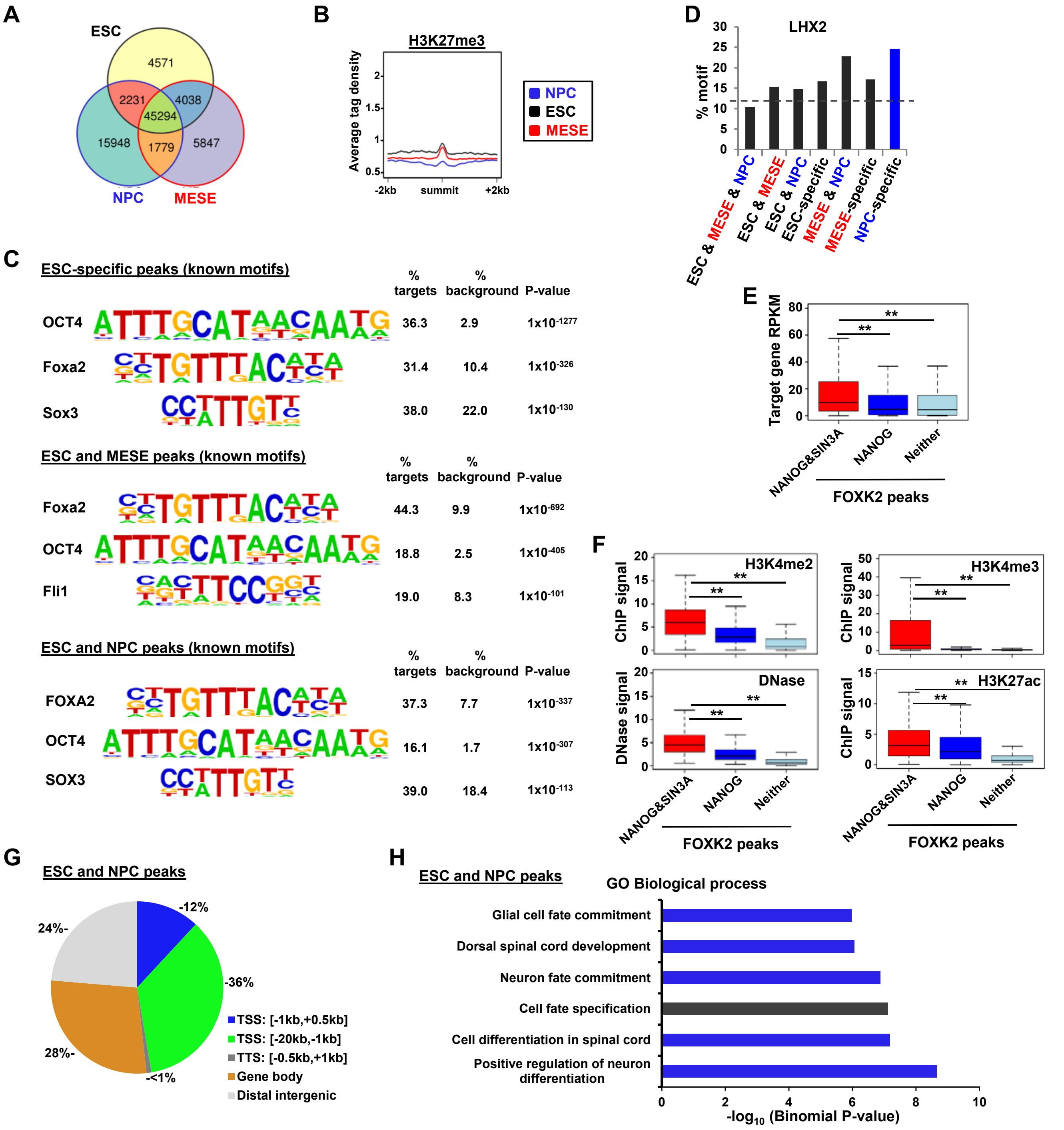

Supplementary Figure S11. Analysis of ChIP-seq data of FOXK2 binding in ESCs, NPCs, MESE cells. (A) Venn diagram showing the overlap of FOXK2 binding regions in ESCs, mesendodermal (MESE) cells and neuronal precursor cells (NPCs). (B) Average tag density plots of H3K27me3 surrounding the summits (plus/minus 2 kb) of the NPC-specific FOXK2 binding peaks. Data are shown for histone marks from NPCs (blue), ESCs (black) or MESE cells (red). (C) DNA binding motif discovery in ESC-specific, ESC&MESE and ESC&NPC FOXK2 binding regions (+/- 200 bp from peak summit). (D) Enrichment of the LHX2 motif within each particular FOXK2 binding peak category (top 20% of peaks are used in each case). The percentage of peaks containing the motif are shown and the dotted line is the average across randomly selected genomic regions. (E) Box plot showing the levels of target genes (defined as closest gene to the peak) activity (RPKM) in ESCs. Horizontal lines on the boxes represent median expression; **= p-value <<0.001.(F) Boxplots of DNase or ChIP-seq signals (RPKM) for the indicated chromatin marks at regions bound by FOXK2 alone or co-bound with NANOG or with both NANOG and SIN3A. **= p-value <<0.001. (G)Distributions of ESC&NPC FOXK2 binding regions across different genomic locations. (H) Gene Ontology categories enriched in genes associated with ESC&NPC FOXK2 peaks.

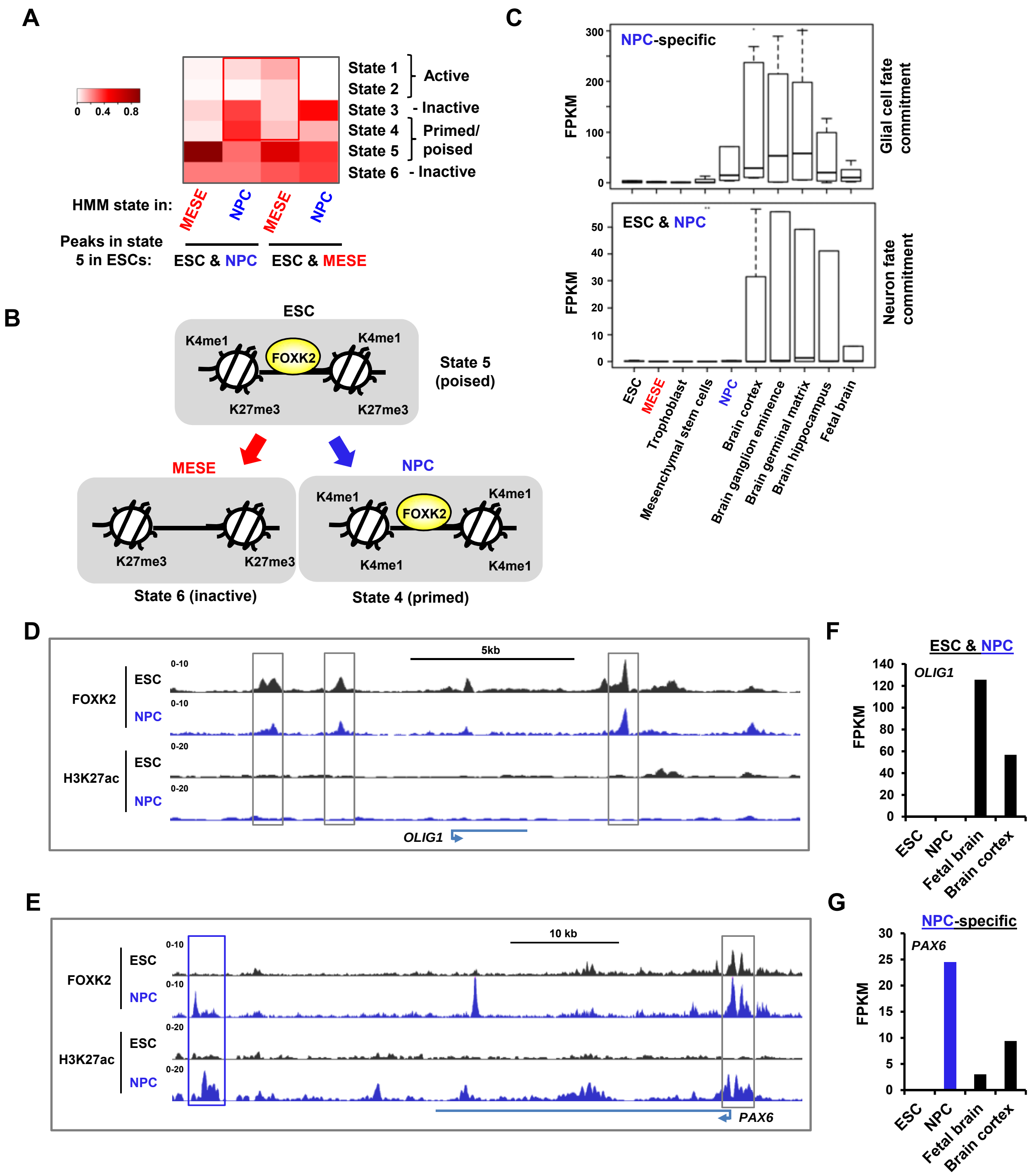

Supplementary Figure S12. Regulatory dynamics of FOXK2 binding regions. (A) HMM states in NPCs or MESE cells for the indicated classes of FOXK2-bound regions (ESC&NPC or ESC&MESE) that exist in state 5 (ie "poised enhancers") in ESCs. (B) Model illustrating the chromatin state changes associated with ESC&NPC FOXK2 binding regions from ESCs to NPCs or MESE cells. Only the predominant state change in each case is illustrated. (C) Boxplots of the expression of the FOXK2 target genes contained in the indicated GO term categories (right) across the indicated cell types/tissues. Genes associated with either NPC-specific FOXK2 binding regions (top) or ESC&NPC FOXK2 binding regions (bottom) were analysed. (D and E) UCSC genome browser views of FOXK2 and H3K27ac ChIP-seq binding profiles in ESCs and NPCs for examples of ESC&NPC FOXK2 binding (*OLIG1*; D) or NPC-specific FOXK2 binding (*PAX6*; E). Cell type-specific (blue) and shared (grey) regions bound by FOXK2 are boxed. (F and G) Examples of the cell/tissue-specific expression of genes associated with ESC&NPC FOXK2 (*OLIG1*; E) or NPC-specific (*PAX6*; F) binding regions are shown.

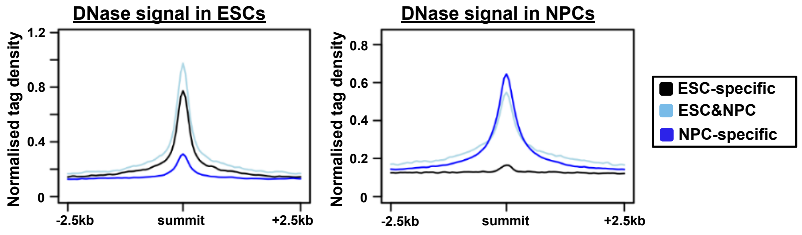

Supplementary Figure S13. Dynamic chromatin changes in NPC-associated FOXK2 binding events. Changes to open chromatin. The average tag density of DNase-seq signal from ESCs (left) or NPCs (right) surrounding the summits of the ESC-specific, ESC&NPC or the NPC-specific FOXK2 binding peaks.

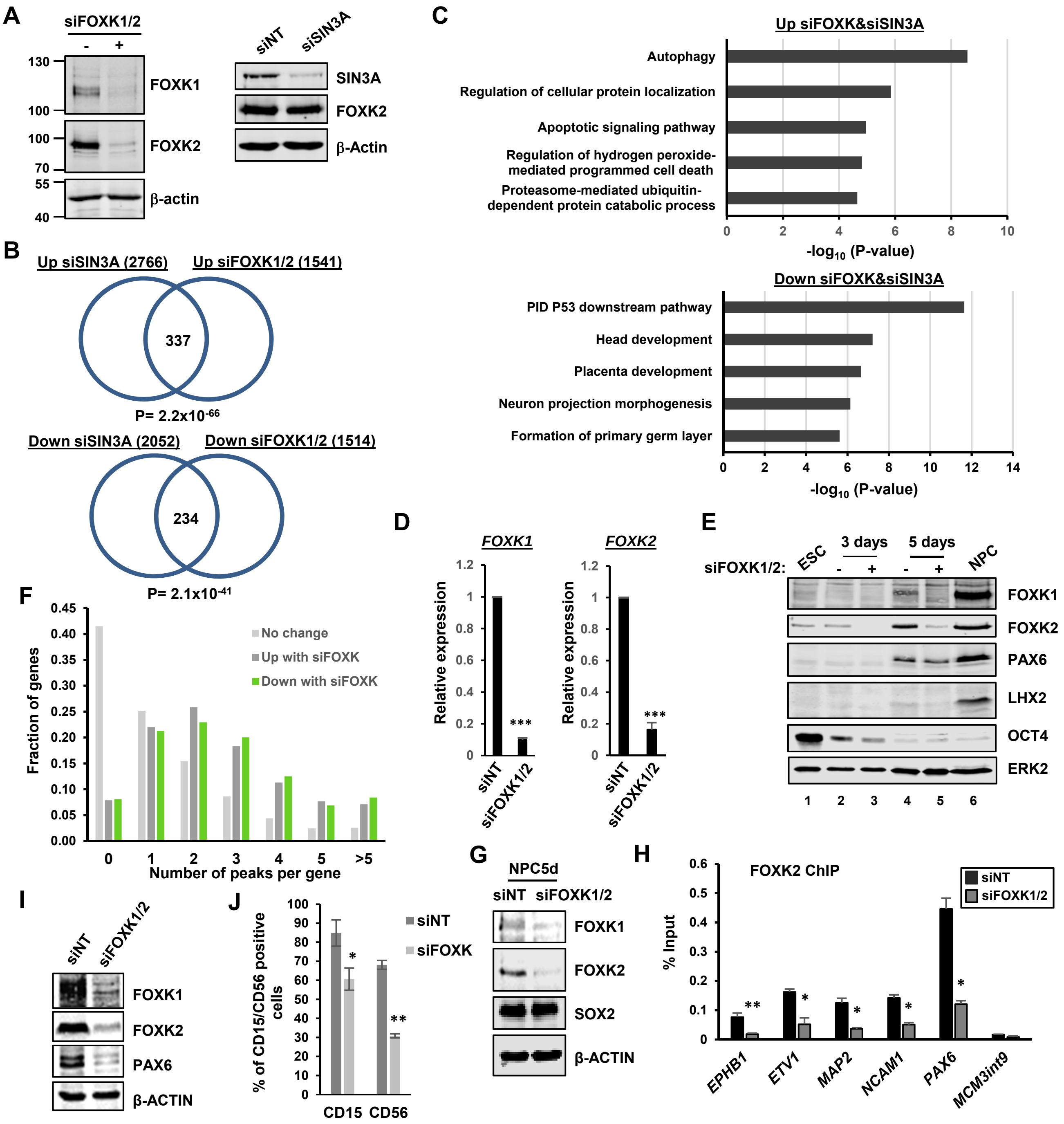

Supplementary Figure S14. Gene regulatory effects of FOXK2 depletion. (A) Western blot of FOXK1/2 and SIN3A expression in one of the samples sent for RNAseq analysis following FOXK1/2 depletion (left) or SIN3A depletion (right). (B) Venn diagram showing the overlap of genes upregulated (top) or down regulated (bottom) by either FOXK1/2 or SIN3A depletion (P value < 0.01 and averaged RPKM >1). The significance of the overlaps is shown (hypergeometric test). (C) Top 5 GO terms associated with genes commonly up (top) or down (bottom) regulated following depletion of SIN3A or FOXK1/2. (D) RT-PCR analysis of *FOXK1* and *FOXK2* expression following 5 days differentiation towards NPCs in the presence of siFOXK1/2 (n=3; ***=P-value <0.001). (E) Western blot analysis of the expression of FOXK1/2 and several NPC marker proteins during the differentiation of ESCs to NPCs over a 7 day period in the presence of control siRNAs (-) or siRNAs against FOXK1 and FOXK2 (+). Undifferentiated ESCs and fully differentiated NPCs are shown as controls. (F) Fraction of genes in the indicated datasets (up/down in ESCs with FOXK1/2 depletion or unchanged) containing the indicated numbers of peaks (defined as peaks within 10 kb of either direction from the TSS). (G and I) Western blots of FOXK1/2 expression in one of the samples used for ChIP analysis in (H) and cell surface marker detection in (J). (H) ChIP analysis of FOXK2 binding to FOXK2 bound regions associated with the indicated genes following FOXK1/2 depletion or non-targeting (NT) siRNA treatment during NPC differentiation for 5 days. Data are shown as SEM; n=3). Asterisks refer to P-values; *= <0.05, **= <0.01. (J) Numbers of cells expressing the NPC marker proteins CD15 (SSEA1) or CD56 (NCAM1) following 8 days differentiation of ESCs to NPCs in presence of non-targeting (NT) control or siFOXK1/2 siRNAs (n=3; * and **=P-values <0.05 and <0.01).
