# Supplementary material for: The forkhead transcription factor FOXK2 premarks lineage-specific genes in human embryonic stem cells for activation during differentiation
Source: Nucleic Acids Res. 2021 Jan 12;49(3):1345–63. doi: 10.1093/nar/gkaa1281 (PMC7897486; doi:10.1093/nar/gkaa1281)
Supplement: gkaa1281_Supplemental_Files [file gkaa1281_supplemental_files.zip › Supplementary Table 1- CRISPR reagents.pdf]

Supplementary Table S1. CRISPR reagents

Oligos and gBlock DNA sequence used to create and validate the FOXK2-DHFR cell line.

Oligonucleotides:

NAME	APPLICATION	SEQUENCE	ADS#
FOXK2-DHFR-Puro gBlock	homology-directed repair (HDR) donor template	Sequence with colour index listed below	---
FOXK2_ sgRNAb_F	Oligos for gRNA	5’-caccGGGTGTCCAGAACTAGCGACC-3’	ADS5907
FOXK2_ sgRNAb_R	Oligos for gRNA	5’-aaacGGTCGCTAGTTCTGGACACCC-3’	ADS5908
FOXK2_ sgRNAc_F	Oligos for gRNA	5’-caccGCTCTCCCGGTCGCTAGTTC-3’	ADS6309
FOXK2_ sgRNAc_R	Oligos for gRNA	5’-aaacGAACTAGCGACCGGGAGAGC-3’	ADS6310
PX458_seq	Sequencing PX458 FOXK2 gRNA plasmids	5’-TTTATGGCGAGGCGGCGG-3’	ADS5909
U6Fwd	Sequencing PX458 FOXK2 gRNA plasmids	5’-GAGGGCCTATTTCCCATGATTCC-3’	ADS5216
FOXK2DHFRHRV_F1	PCR genotyping	5’-CCGCTAATCAGGCAGTTCAC-3’	ADS6306
FOXK2-DHFR-seqR1	PCR genotyping	5’-CTGTGAGAGTTCTGCGCATC-3’	ADS6334
FOXK2DHFRHRV_F2	PCR genotyping	5’-CTTTGAGATTCTGGAGCGGC-3’	ADS6307
FOXK2DHFRHRV_R2	PCR genotyping	5’-TCCCTGGGTTCTCAAGGTTC-3’	ADS6308

gBlock sequence:

```
AGCCCAGGCTCCTGTGCCCTTGGCCACCGTGGGCCAGTCCCTGGGGTCTTGTGGGAGCCTCCGCGGGCCTGGGAGTGGCA
GGACCCTTAGAGGGCGAGAGTTCACATGAGAGCGTGGGGTTCTGACTCGCGAGGGTTCACGTGAGAGCGTGGGGTTCTGA
GTCGCGAGGGTTCACGTGAGAGCGTGGGGTTCTGACTCCCTCGTGTCATTTCAGCCGCGGCGAGTCCTTTGCACATGTTG
GCAACACACGCATCCGCATCGGCCTCCCTGCCCACAAAGCGCCACAACGGTGACCAGCCGGAGCAGCCGGAGCTGAAGCG
GATCAAGACAGAAGACGGCGAGGGCATCGTCATTGCCCTGAGCGTGGACACGCCACCGGCAGCCGTAAGGGAAAAGGGTG
TCCAGAACggaggcggttacccatacgatgttcctgactatgcgggctatccctatgacgtcccggactatgcaggatcc
tatccatatgacgttccagattacgctgatatcatgatcagtctgattgcggcgttagcggtagattacgttatcggcAT
GGAAAACGCCATGCCGTGGAACCTGCCTGCCGATCTCGCCTGGTTTAAACGCAACACCTTAAATAAACCCGTGATTATGG
GCCGCCATACCTGGGAATCAATCGGTCGTCCGTTGCCAGGACGCAAAAATATTATCCTCAGCAGTCAACCGAGTACGGAC
GATCGCGTAACGTGGGTGAAGTCGGTGGATGAAGCCATCGCGGCGTGTGGTGACGTACCAGAAATCATGGTGATTGGCGG
CGGTCGCGTTATTGAACAGTTCTTGCCAAAAGCGCAAAAACTGTATCTGACGCATATCGACGCAGAAGTGGAAGGCGACA
CCCATTTCCCGGATTACGAGCCGGATGACTGGGAATCGGTATTCAGCGAATTCCACGATGCTGATGCGCAGAACTCTCAC
AGCTATTGCTTTGAGATTCTGGAGCGGCGAaagcttgagggcagaggaagtcttctaacatgcggtgacgtggaggagaa
tcccggccctgctagcggtagcggcagcggtagcatgaccgagtacaagcccacggtgcgcctcgccacccgcgacgacg
tcccccgggccgtacgcaccctcgccgccgcgttcgccgactaccccgccacgcgccacaccgtcgacccggaccgccac
atcgagcgggtcaccgagctgcaagaactcttcctcacgcgcgtcgggctcgacatcggcaaggtgtgggtcgcggacga
cggcgccgcggtggcggtctggaccacgccggagagcgtcgaagcgggggcggtgttcgccgagatcggcccgcgcatgg
ccgagttgagcggttcccggctggccgcgcagcaacagatggaaggcctcctggcgccgcaccggcccaaggagcccgcg
tggttcctggccaccgtcggcgtctcgcccgaccaccagggcaagggtctgggcagcgccgtcgtgctccccggagtgga
ggcggccgagcgcgccggggtgcccgccttcctggagacctccgcgccccgcaacctccccttctacgagcggctcggct
tcaccgtcaccgccgacgtcgaggtgcccgaaggaccgcgcacctggtgcatgacccgcaagcccggtgcctgacgaccg
ggagagcttttctttaacgatatcaactctgtggtgccaaaaggagacgcggcctcccgccagcactcgggggtgcaggg
ccctgtggttggacttcacctctcagcactgaaaacccaaaacccagctggccttaacactccttaaagacagaagtcac
acttgaacaaaacccacacacaacaaaacctgatttgggagacggtgtctccactgagcacctgctgggctgagcttcta
cctacgagtgaaactctgtcctcccgcgaggaccaggcatcgctgtgtgaggacggcacggccagcgcctgctgtgagtg
```

Left homology arm
Right homology arm

Dihydrofolate reductase type I [Escherichia coli]
N2Askip
Puromycin resistance protein coding region
