# Supplementary material for: The forkhead transcription factor FOXK2 premarks lineage-specific genes in human embryonic stem cells for activation during differentiation
Source: Nucleic Acids Res. 2021 Jan 12;49(3):1345–63. doi: 10.1093/nar/gkaa1281 (PMC7897486; doi:10.1093/nar/gkaa1281)
Supplement: gkaa1281_Supplemental_Files [file gkaa1281_supplemental_files.zip › Supplementary Table S2- Antibodies used in this study.docx]

| PRIMARY ANTIBODIES | SUPPLIER | SPECIES | TYPE | APPLICATION | REFERENCE |
| --- | --- | --- | --- | --- | --- |
| FOXK1 | Bethyl Laboratories | Rabbit | Polyclonal | WB | A301-728A |
| FOXK2 | Abcam | Goat | Polyclonal | WB | ab5298 |
| FOXK2 | Bethyl Laboratories | Rabbit | Polyclonal | WB, IP, ChIP | A301-729A |
| BAP1 | Santa Cruz | Mouse | monoclonal | WB | C-4, SC-28383 |
| SIN3A | Novus Biologicals | Rabbit | Polyclonal | WB | NB600-1263 |
| HDAC1 | Abcam | Rabbit | Polyclonal | WB | ab7028 |
| SOX2 | Abcam | Rabbit | Polyclonal | WB | ab97959 |
| NANOG | Abcam | Rabbit | Polyclonal | WB | ab21624 |
| OCT4 | Abcam | Rabbit | Polyclonal | WB | ab19857 |
| EOMES | Abcam | Rabbit | Polyclonal | WB | ab23345 |
| T (Brachyury) | R&D Systems | Goat | Polyclonal | WB | AF2085 |
| PAX6 | Millipore | Rabbit | Polyclonal | WB | AB2237 |
| LHX2 | Santa Cruz | Goat | Polyclonal | WB | Sc-19344 |
| SOX9 | Millipore | Rabbit | Polyclonal | WB | AB5535 |
| LAMIN B | Santa Cruz | Goat | Polyclonal | WB | Sc-6216 |
| ERK2 | Santa Cruz | Rabbit | Polyclonal | WB | sc-154 |
| β-ACTIN | Millipore | Mouse | monoclonal | WB | MAB1501 |

**Supplementary Table S2. List of antibodies.** Applications listed are Western blot (WB), immunoprecipitation (IP), and chromatin IP (ChIP).
