# Supplementary material for: The forkhead transcription factor FOXK2 premarks lineage-specific genes in human embryonic stem cells for activation during differentiation
Source: Nucleic Acids Res. 2021 Jan 12;49(3):1345–63. doi: 10.1093/nar/gkaa1281 (PMC7897486; doi:10.1093/nar/gkaa1281)
Supplement: gkaa1281_Supplemental_Files [file gkaa1281_supplemental_files.zip › Supplementary Table S3- PCR primers_revised.pdf]

Name	ADS#		SEQUENCE (5'-3')
RT_FOXK1	ADS1372<br>ADS1373	FORWARD PRIMER<br>REVERSE PRIMER	CGAGTTCGAGTTCCTCATGC<br>GGGAGATCTGGGGGTACAGT
RT_FOXK2	ADS1745<br>ADS1746	FORWARD PRIMER<br>REVERSE PRIMER	GCTGACAACTCACAGCCTGA<br>TCCGCAGTCCTGTAGTAGGG
RT_SIN3A	ADS1642<br>ADS1643	FORWARD PRIMER<br>REVERSE PRIMER	GCACAGAGGCTTTTCCTCAC<br>TGATGGCTGCTATGAACTGC
RT_GAPDH	ADS2184<br>ADS2185	FORWARD PRIMER<br>REVERSE PRIMER	ACAGTCAGCCGCATCTTCTT<br>TTGATTTTGGAGGGATCTCG
RT_HMBS	ADS2858<br>ADS2859	FORWARD PRIMER<br>REVERSE PRIMER	GAGAAGAATGAAGTGGACCT<br>GAAAGACAACAGCATCATGAG
RT_OCT4	ADS4792<br>ADS4793	FORWARD PRIMER<br>REVERSE PRIMER	CCTTCGCAAGCCCTCATTTC<br>TAGCCAGGTCCGAGGATCAA
RT_NANOG	ADS4794<br>ADS4795	FORWARD PRIMER<br>REVERSE PRIMER	ATAACCTTGGCTGCCGTCTC<br>AGCCTCCCAATCCCAAACAA
RT_SOX2	ADS4796<br>ADS4797	FORWARD PRIMER<br>REVERSE PRIMER	ATGGACAGTTACGCGCACAT<br>CGAGCTGGTCATGGAGTTGT
RT_DNMT3B	ADS4798<br>ADS4799	FORWARD PRIMER<br>REVERSE PRIMER	GCCCTGGAGACTCATTGGAG<br>CACGACGCACCTTCGACTTA
RT_KLF4	ADS4800<br>ADS4801	FORWARD PRIMER<br>REVERSE PRIMER	TACCAAGAGCTCATGCCACC<br>GGTGTGCCTTGAGATGGGAA
RT_T (brachyury)	ADS5292<br>ADS5293	FORWARD PRIMER<br>REVERSE PRIMER	ACCCAGTTCATAGCGGTGAC<br>CCATTGGGAGTACCCAGGTT
RT_EOMES	ADS5296<br>ADS5297	FORWARD PRIMER<br>REVERSE PRIMER	TCCACCTTGATGCATCCTGT<br>CCAATGTTCACAGCCTGCTT
RT_SOX17	ADS5300<br>ADS5301	FORWARD PRIMER<br>REVERSE PRIMER	GGACCGCACGGAATTTGAAC<br>GGATCAGGGACCTGTCACAC
RT_WNT3	ADS5294<br>ADS5295	FORWARD PRIMER<br>REVERSE PRIMER	GACTTCGGCGTGTTAGTGTC<br>TGTGGTCCAGGATAGTCGTG
RT_MIXL1	ADS5290<br>ADS5291	FORWARD PRIMER<br>REVERSE PRIMER	TTCCATTGGTCTGCATCCCT<br>AGAGACGGGGTAGAGTGACT
RT_CGA	ADS5306<br>ADS5307	FORWARD PRIMER<br>REVERSE PRIMER	ATGCACGCTACAGGAAAACC<br>CCCCATTACTGTGACCCTGT
RT_FOXG1	ADS5980<br>ADS5981	FORWARD PRIMER<br>REVERSE PRIMER	TGGGACCTACTCCCTCAACC<br>TCCCGTCGTAAAACTTGGCA
RT_LHX2	ADS5978<br>ADS5979	FORWARD PRIMER<br>REVERSE PRIMER	AAGTTCAGGCGCAACCTCTT<br>AAGACGGACGTCACAGTTGG
RT_SOX1	ADS6321<br>ADS6322	FORWARD PRIMER<br>REVERSE PRIMER	AGGCCATGGATGAAGGACAA<br>TTTGCCCGTTTTCCCAAGAG
RT_SOX9	ADS6319<br>ADS6320	FORWARD PRIMER<br>REVERSE PRIMER	GAGGAAGTCGGTGAAGAACGG<br>CCTCTCGCTTCAGGTCAGC
RT_NES	ADS5984<br>ADS5985	FORWARD PRIMER<br>REVERSE PRIMER	GTAGCTCCCAGAGAGGGGAA<br>CTCTAGAGGGCCAGGGACTT
RT_PAX6	ADS5982<br>ADS5983	FORWARD PRIMER<br>REVERSE PRIMER	TTGCAGCCTACATTCCCTGA<br>GGGTCTCCAAAGTCTCTGCT
RT_OTX2	ADS5491<br>ADS5492	FORWARD PRIMER<br>REVERSE PRIMER	CCCACTGTCAGATCCCTTGT<br>GGAAAGAGAAGCTGGGGACT
ChIP_EPHB1	ADS6793<br>ADS6794	FORWARD PRIMER<br>REVERSE PRIMER	AGCCACATTCAATCGACAGC<br>GTGCTTGGCTGGTCACTATG

ChIP_ETV1	ADS6795<br>ADS6796	FORWARD PRIMER<br>REVERSE PRIMER	CACTGTGGGGCTGAAATGAC<br>GGTCCTGCTTGCAAGTAACC
ChIP_MAP2	ADS6797<br>ADS6798	FORWARD PRIMER<br>REVERSE PRIMER	TCCTTCCTCCATTCCGACTG<br>AGTCAGTGGCCATGCAATTC
ChIP_NCAM1	ADS6799<br>ADS6800	FORWARD PRIMER<br>REVERSE PRIMER	GAACTGATTGGGCGACATCC<br>ATGTGGAGGGTGAACGAGAG
ChIP_PAX6	ADS6801<br>ADS6802	FORWARD PRIMER<br>REVERSE PRIMER	TGTTAATGTGTGTGTGCCGG<br>ATTTTGTGTGAGAGCGAGCG
ChIP_MCM3int9	ADS2387<br>ADS2388	FORWARD PRIMER<br>REVERSE PRIMER	ACTTTCCAGGGGATTCTGCT<br>TTGCTGACTAACTGGGCTGA

Supplementary Table S3. **Oligonucleotide primers used in RT-qPCR and ChIP-qPCR**.
